# A Robust Reactive Static Obstacle Avoidance System for Surface Marine Vehicles

**DOI:** 10.3390/s20216262

**Published:** 2020-11-03

**Authors:** Rafael Guardeño, Manuel J. López, Jesús Sánchez, Alberto González, Agustín Consegliere

**Affiliations:** 1 Escuela Superior de Ingeniería, Universidad de Cádiz, 11519 Puerto Real, Spain; manueljesus.lopez@uca.es (M.J.L.); agustin.consegliere@uca.es (A.C.); 2 Sistemas, Navantia, 11100 San Fernando, Spain; jsanchezpa@navantia.es; 3 Smart Sustainment, Navantia, Sydneyn NSW 2000, Australia; agonzalezc@navantia.es

**Keywords:** unmanned surface vehicle, autonomous navigation, static obstacle avoidance, LIDAR sensor modeling, occupancy probability grid, exponential discretization, estimated closed-loop model, repulsive forces, random scenario generation, unknown and congested scenarios

## Abstract

This paper is centered on the guidance systems used to increase the autonomy of unmanned surface vehicles (USVs). The new Robust Reactive Static Obstacle Avoidance System (RRSOAS) has been specifically designed for USVs. This algorithm is easily applicable, since previous knowledge of the USV mathematical model and its controllers is not needed. Instead, a new estimated closed-loop model (ECLM) is proposed and used to estimate possible future trajectories. Furthermore, the prediction errors (due to the uncertainty present in the ECLM) are taken into account by modeling the USV’s shape as a time-varying ellipse. Additionally, in order to decrease the computation time, we propose to use a variable prediction horizon and an exponential resolution to discretize the decision space. As environmental model an occupancy probability grid is used, which is updated with the measurements generated by a LIDAR sensor model. Finally, the new RRSOAS is compared with other SOA (static obstacle avoidance) methods. In addition, a robustness study was carried out over a set of random scenarios. The results obtained through numerical simulations indicate that RRSOAS is robust to unknown and congested scenarios in the presence of disturbances, while offering competitive performance with respect to other SOA methods.

## 1. Introduction

**Figure 1:**
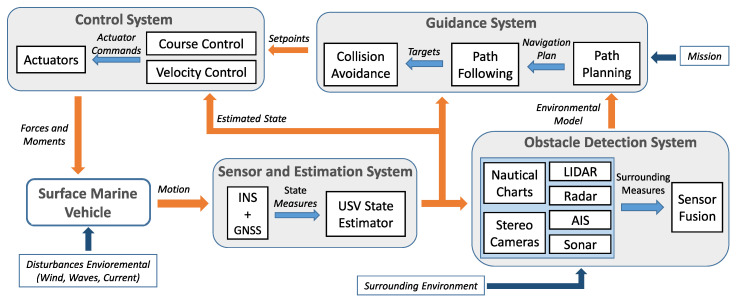
Interaction between the systems of an unmanned surface marine vehicle [31].

Nowadays, the application fields of unmanned surface vehicles (USVs) are very diverse [1], such as environmental control, scientific activities, commercial work, national security issues and surveillance. Consequently, the systems that provide the capacities required in order to consider a vessel as autonomous have become a very active field of research [1,2,3]. These systems, which are shown in Figure 1, must: estimate the vessel’s state vector [4,5], control its course and velocity through the actuators [4,5], process the sensor measurements to generate a model of the environment surrounding the USV [6,7,8,9,10,11] and carry out safe guidance of the vehicle towards its goal [3,4,5,7,9,12,13,14,15,16,17,18,19,20,21,22]. Therefore, the autonomy of a USV is determined by the level of development of the estimation, control, obstacle detection and guidance systems, and by their integration in a hardware platform [1,2,3]. More specifically, a USV’s guidance system is formed by three main subsystems: path planning, path following and obstacle avoidance (reactive algorithms). First, planning algorithms [3,7,18,23,24] generate collision-free paths with known obstacles that, in turn, lead the vehicle to its goal. Once a path is established, the path following algorithms change the course and velocity of the USV to follow it [4,5,19,25]. Finally, due to the presence of unknown obstacles for the path planner, reactive algorithms use the information provided by the obstacle detection system (generated from sensors’ measurements) to modify the course/velocity setpoints in order to avoid a collision [9,12,13,14,15,16,26,27,28,29,30]. Of the systems shown in Figure 1, this work is focused on the obstacle avoidance (OA) systems applied to the USV.

### 1.1. Overview of Reactive Algorithms Applied to USVs

Today there are many reactive methods that provide a vessel with the capacity to avoid obstacles [9,11,13,14,15,16,17,23,25,32,33,34,35,36,37,38,39,40,41,42,43,44,45,46,47,48]. To illustrate this diversity, while the great majority of these methods have been designed for propulsion vessels, the approach proposed in [48] uses a potential reactive field to take into account the kinematic limitations of a sailboat due to wind direction. In the case of propelled vessels, most of these works propose dynamic obstacle avoidance (DOA) systems for situations in open sea [9,13,14,15,16,17,23,33,34,35,36,37,38,39,40,44,45,46,47]. Although some of them also consider a specific approach for static obstacles [16,17,23,34,35,37,40,45,46], their performances and robustness have not been evaluated in depth. As a minor proportion, works such as [11,25,32,41,42,43], propose static obstacle avoidance (SOA) systems for USVs. In addition to the classification based on the kind of obstacle, AO systems for USVs can also be categorized depending on the used approach. Firstly, there is a wide variety of OA systems [11,13,14,32,35,38,40,41,42,43,45,48] that use or modify reactive methods proposed for mobile robots [26,27,28,30,49,50,51,52,53,54]. Specifically, in [32,35,38,42,45,48] OA systems are based on the potential fields approach [28,49,50]. Alternatively, in [11,40,41,43] the authors adapted the dynamic window [27] and the VFH+ (vector field histogram plus, [30,51]), which they used as SOA systems for USVs. In addition, a collision cone [54] was also used in [40] to avoid dynamic obstacles. One of the DOA methods from mobile robotics most applied to USVs [13,14,44] is velocity obstacles [26] (it has variants [52,53]). On the other hand, in works such as [23,33,37,47,55,56] the OA systems are based on modifications of path planning algorithms like A* [7]. Another important approach was used in [9,15], whose OA systems carried out the fuzzification of target vessel variables through membership functions. Finally, a commonly used approach is based on predicting the possible future paths that the USV could follow if a collision risk were to appear [16,17,34,36,39,40,44,46].

### 1.2. Related Works

First, before reading this work, we recommended that the reader consults in detail the paper [31], where a new autotuning environment is proposed for static obstacle avoidance methods applied to USVs. Specifically, to develop and evaluate the RRSOAS, the numerical simulation environment proposed in [31] was used. Moreover, several reactive methods [30,35,41,42] were autotuned in that paper, and later used in this work to make a comparison with the RRSOAS.

Due to the intensive research on obstacle avoidance methods applied to mobile robots, there are reactive algorithms that can guarantee the safety of these systems [12,20,21,22,26,27,30]. For instance, the approach proposed in [22] for an underwater robot is based on two behavioral modules: avoiding obstacles and seeking the goal, which are constituted by two ANFISs (adaptive neuro fuzzy inference systems) that enable the robot’s motion in vertical and horizontal planes. Another important approach is proposed in [21], where from a tangent graph (that considers the robot’s minimum turning radius) the authors define a probabilistic algorithm that ensures safe navigation to the goal in 100% of cases. In view of these results, the proposal of new reactive algorithms for USVs is justified due to the disturbances inherent to the marine environment [4], and the dynamics and establishment times characteristic of marine surface vehicles [5,57,58,59,60]. For these reasons, the reactive algorithm proposed in this work, in line with the approach followed in [16,17,34,36,39,40,44,46], takes into account the USV’s dynamics when it requests any change in the control setpoints. In case of a possible collision, OA systems study a set of alternative setpoints for the course and velocity for the vehicle [12,20]. This set, or decision space (DS), contains infinite course/velocity setpoints, so it can be discretized for study. In works such as [11,13,14,16,17,26,27,30,40,41,43], the discrete decision spaces (DDS) are obtained from fixed resolutions. As a novel contribution, we propose to discretize the SD by applying an exponential resolution for the course’s setpoints. In this way, a variable resolution is achieved, high in the vicinity of the current course and low in the limits of DS, which reduces the number of alternative setpoints. Furthermore, to take into account the vessel’s dynamics, it is common to make predictions of the possible paths that the USV would follow if the course/velocity setpoints were modified. For this purpose, in [16,17,34,36,39,40,44,46] it is necessary to know the mathematical modeling of the vessel and its controllers. In contrast with these methods, RRSOAS does not need these models. Instead, we propose a new estimated closed-loop system model (ECLM) to estimate possible future paths. This ECLM simplifies the dynamics of the closed-loop system under the hypothesis of a correct operation of the course/speed controllers, and in turn, considers the characteristic dynamics of marine surface vehicles [5,57,58,59,60]. With respect to the prediction horizon that defines the limit of these future paths, the majority of the reactive algorithms for USVs [16,17,34,36,39,44,46] use a fixed horizon. As a result, depending mainly on the velocity setpoint, these paths cover different distances for the same prediction time. In order to reduce the runtimes of these predictions, this paper proposes to use a variable prediction horizon. In [61] the prediction horizon is defined as a variable that is optimized by a predictive controller designed for unmanned aerial vehicles [62]. Nevertheless, in our work the prediction horizon depends on each pair of alternative setpoints; thus, all possible future paths cover the same distance. Moreover, the uncertainty present in the mathematical model used to make the predictions is not taken into account in [16,17,34,36,39,40,44,46]. This uncertainty, due to the recursive calculation of the predictions, causes an error that increases with the prediction time. In order to make the RRSOAS robust to these prediction errors, we propose to model the USV contour as an ellipse whose perimeter varies with the prediction step. Thus, the shape associated with the vehicle increases with the prediction error. Finally, RRSOAS uses an occupation grid (OG) as a model of the environment [6], which must be generated by an obstacle detection system. OGs have been widely used in mobile robotics [6,7,8,29] and in guidance systems for USVs [23,24,33,37,47]. As a novel contribution, in contrast to the general approach in which the predictions are evaluated for each obstacle [16,17,34,36,39,40,44,46], we propose to translate the possible future paths to an occupancy probability grid. For this purpose, the concept of repulsive forces proposed in [29] is adapted and an estimated collision time based on the probability of occupation is proposed. In this way, taking into account the USV’s dynamics, RRSOAS considers the measurement/estimation errors present in the environment model. In reactive algorithms, it is very common to use a heuristic to determine the control commands requested to the autonomous vehicles [11,12,13,14,16,17,20,26,27,30,33,40,41,44,46,47]. Therefore, once the alternative setpoints have been characterized according to a repulsive force and an estimated collision time, both associated with each future path, a heuristic is employed to calculate the course and velocity setpoints demanded by the USV controllers.

The organization of this paper is as follows: mathematical models used to evaluate the new RRSOAS by means of numerical simulations are described in the Section 2. As a simulation environment (USV and LIDAR sensor models), the one proposed in [31] is used. In addition, a Bayesian filter [8] is employed to generate the occupancy probability grids at each sample time of the reactive algorithm [63]. On the other hand, Section 3 presents in detail the four subsystems that form the RRSOAS. As results, the RRSOAS’s performance is compared with those of other SOA methods [30,31,41] in Section 4. Furthermore, a study of the robustness of the algorithm was also carried out on nine-hundred obstacle avoidance scenarios, in which the USV navigates at different velocities and is affected by several levels of sea current. Finally, Section 5 contains a general discussion of the results, and in Section 6 the conclusions are presented.

## 2. Mathematical Models

This section describes the mathematical models used to evaluate the RRSOAS by means of numerical simulations.

### 2.1. USV Model

**Figure 2:**
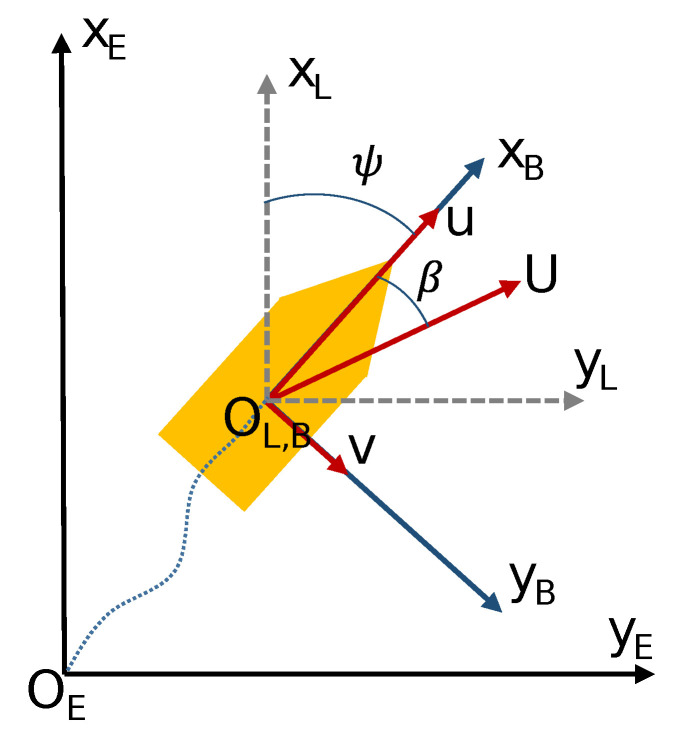
Earth, local and body axis reference systems adopted in this work, where β is the slip angle.

As in most OA methods applied to USVs [14,15,16,17,25,33,34,35,38,39,40,41,43,44,45,46,47], the algorithm proposed in this work was evaluated through numerical simulations with a three-degrees of freedom vessel model. Furthermore, in line with the general approach followed in this field [11,13,14,16,33,34,35,36,37,38,39,41,42,43], the RRSOAS uses course and velocity controllers to govern the vessel. In particular, the modeling proposed in [31] for a USV of 9.2 m of length is used. Compared with other USV’s models [34,38,40,64,65], this modeling takes into account vessel model (1), current effect (2) in terms of relative velocity, actuator model (3) and course/velocity controllers (5). A complete description of these models and the numerical values of their parameters is available in [31].

(1) $$\begin{array}{cc} \; & \dot{u}\left(m-X_{\dot{u}}\right)=mvr+mx_{g}r^{2}-Y_{\dot{v}}v_{r}r+X_{u}u_{r}+X_{|u|u}\left|u_{r}\right|u_{r}+\tau_{x}\\ \; & \dot{v}\left(m-Y_{\dot{v}}\right)+mx_{g}\dot{r}=-mur-X_{\dot{u}}u_{r}r+Y_{v}v_{r}+Y_{r}r+Y_{|v|v}v_{r}|v_{r}|+Y_{|r|v}\left|r\right|v_{r}+Y_{|v|r}\left|v_{r}\right|r+\tau_{y}\\ \; & \dot{r}\left(I_{z}-N_{\dot{r}}\right)+mx_{g}\dot{v}=-mx_{g}ur-N_{uv}v_{r}u_{r}+N_{r}r+N_{v}v_{r}+N_{|v|r}|v_{r}|r+N_{|r|v}\left|r\right|v_{r}+N_{|r|r}\left|r\right|r+\tau_{n}\\ \; & \underset{\dot{\eta }}{\underset{︸}{\left(\begin{array}{c} {\dot{x}}_{E}\\ {\dot{y}}_{E}\\ \dot{\psi } \end{array}\right)}}=\underset{R(\psi )}{\underset{︸}{\left(\begin{array}{ccc} cos(\psi ) & -sin(\psi ) & 0\\ sin(\psi ) & cos(\psi ) & 0\\ 0 & 0 & 1 \end{array}\right)}}\underset{\nu }{\underset{︸}{\left(\begin{array}{c} u\\ v\\ r \end{array}\right)}} \end{array}$$

where η represents the position vector of the USV referring to Earth-axes, ν and $\nu_{r}$ are the velocity and relative velocity vectors referring to body-axes, R(ψ) is the rotation matrix from body-axes to Earth-axes (see Figure 2) and $\tau_{\mathrm{act}}={\left(\tau_{x}\;\tau_{y}\;\tau_{z}\right)}^{T}$ are the forces and moment applied by the actuators.

(2) $$\nu_{r}={\left(u_{r}\;v_{r}\;r\right)}^{T}={\left(\begin{array}{ccc} \left(u-V_{c}cos\left(\beta_{c}-\psi \right)\right) & \left(v-V_{c}sin\left(\beta_{c}-\psi \right)\right) & r \end{array}\right)}^{T}$$

with $V_{c}$ as the module of the current velocity and $\beta_{c}$ as its direction.

(3) $$\tau_{\mathrm{act}}=\left(\begin{array}{c} \tau_{x}\\ \tau_{y}\\ \tau_{n} \end{array}\right)=\left(\begin{array}{cc} 1 & 0\\ 0 & 1\\ 0 & -l_{x} \end{array}\right)\left(\begin{array}{c} T_{act}-D_{act}\\ L_{act} \end{array}\right),\;\begin{array}{cc} \; & T_{act}=T_{|n|n}|n|n-T_{|n|u}\left|n\right|u_{r}\\ \; & D_{act}=D_{\left|\delta \right|}\left|\delta |\right|u_{r}|u_{r}\\ \; & L_{act}=(L_{\delta }\delta -L_{|\delta |\delta }\left|\delta |\delta )\right|u_{r}|u_{r} \end{array}$$

where $l_{x}$ is the distance from the actuator to the vessel’s center of gravity; *n* represents the propeller angular velocity; δ is the rudder angle; $T_{act}$ represents the thrust generated by the propeller; and finally, $D_{act}$ and $L_{act}$ are the drag and lift forces generated by the rudder, respectively. In addition, the dynamics of the actuator are considered as:

(4) $$\begin{array}{cc} \; & \dot{\delta }=\left(1/\tau_{\delta }\right)\left(\delta_{c}-\delta \right),\;\delta \;\in \;\left[-\delta_{lim},\delta_{lim}\right],\;\dot{\delta }\;\in \;\left[-{\dot{\delta }}_{lim},{\dot{\delta }}_{lim}\right]\\ \; & \dot{n}=\left(1/\tau_{n}\right)\left(n_{c}-n\right),\;n\;\in \;\left[n_{min},n_{max}\right] \end{array}$$

where the time constants $\tau_{\delta }$ and $\tau_{n}$ characterize rudder and propulsion dynamics, while $\delta_{c}$ and $n_{c}$ represent the setpoints for rudder angle and propeller angular velocity, respectively.

The respective discrete time control algorithms are given by

(5) $$\begin{array}{cc} \delta_{c}\left(k\right) & =\underset{\delta_{P}\left(k\right)}{\underset{︸}{K_{P_{\chi }}e_{\chi }\left(k\right)}}+\underset{\delta_{I}\left(k\right)}{\underset{︸}{T_{m}^{c}K_{I_{\chi }}e_{\chi }\left(k\right)+\delta_{I}\left(k-1\right)}}+\underset{\delta_{D}\left(k\right)}{\underset{︸}{\frac{K_{D_{\chi }}}{T_{m}^{c}}\left(e_{f_{\chi }}\left(k\right)-e_{f_{\chi }}\left(k-1\right)\right)}}\\ n_{c}\left(k\right) & =\underset{n_{P}\left(k\right)}{\underset{︸}{K_{P_{U}}e_{U}\left(k\right)}}+\underset{n_{I}\left(k\right)}{\underset{︸}{T_{m}^{c}K_{I_{U}}e_{U}\left(k\right)+n_{I}\left(k-1\right)}}+\underset{n_{D}\left(k\right)}{\underset{︸}{\frac{K_{D_{U}}}{T_{m}^{c}}\left(e_{f_{U}}\left(k\right)-e_{f_{U}}\left(k-1\right)\right)}}\\ e_{f_{i}}\left(k\right) & =\frac{1}{c_{f}+1}\left(e_{i}\left(k\right)+c_{f}e_{f_{i}}\left(k-1\right)\right),\;e_{i}\left(k\right)=sp_{i}\left(k\right)-i\left(k\right),\;i\in \left\{U,\chi \right\} \end{array}$$

where the USV course is defined as χ=ψ+β (see Figure 2), the sample time has been established as $T_{m}^{c}=0.1$ s and $c_{f}=\tau_{f}/T_{m}^{c}$. Moreover, due to the strong non-linearity of model (1), the authors in [31] use the gain scheduling technique [57,66] to make these PID structures adaptive.

### 2.2. Sensor Environment Modeling

In order to achieve a realistic simulation environment, this work uses a 2D model of the Ultra Puck LIDAR sensor proposed in [31]. This sensor model has a measurement range $d_{range}=200$ m and a horizontal field of view $h_{range}=360^{\circ }$. Furthermore, looking for the worst case [67], the lowest angular resolution ($h_{res}=0.4^{\circ }$) and minimum rotation frequency ($f_{m}^{L}=5$ Hz) were fixed in the numerical simulations. As inputs, as stated in [31], the sensor model requires the USV position vector and a set of segments that define the obstacle scenario ($S_{E}$),

(6) $$S_{E}=\overset{n_{O}}{\underset{i_{O}=1}{⋃}}Obst_{E}^{i_{O}},\;Obst_{E}^{i_{O}}=\left\{\begin{array}{c} seg_{1E}^{i_{O}}=\left(x_{1E}^{i_{O}},y_{1E}^{i_{O}},x_{2E}^{i_{O}},y_{2E}^{i_{O}}\right),\;seg_{2E}^{i_{O}}=\left(x_{2E}^{i_{O}},y_{2E}^{i_{O}},x_{3E}^{i_{O}},y_{3E}^{i_{O}}\right),\\ seg_{3E}^{i_{O}}=\left(x_{3E}^{i_{O}},y_{3E}^{i_{O}},x_{4E}^{i_{O}},y_{4E}^{i_{O}}\right),\;seg_{4E}^{i_{O}}=\left(x_{4E}^{i_{O}},y_{4E}^{i_{O}},x_{1E}^{i_{O}},y_{1E}^{i_{O}}\right) \end{array}\right\}$$

with $n_{O}$ as the number of obstacles that form a scenario, where each obstacle $Obst_{E}^{i_{O}}$ is defined as four concatenated segments $seg_{1E\cdots 4E}^{i_{O}}$ of coordinates $x_{1E\cdots 4E}^{i_{O}}$ and $y_{1E\cdots 4E}^{i_{O}}$ referring to Earth-axes.

Next, in [31] the authors use the set of segments $L_{B}$ to model the LIDAR sensor as a set of beams referring to body-axes:

(7) $$\begin{array}{cc} \; & L_{B}=\left\{seg_{B}^{i_{L}}\left(x_{1B}^{i_{L}},y_{1B}^{i_{L}},x_{2B}^{i_{L}},y_{2B}^{i_{L}}\right)\;|\;x_{1B}^{i_{L}}=y_{1B}^{i_{L}}=0,\;x_{2B}^{i_{L}}=cos\left(i_{L}h_{res}\right)d_{range},\;y_{2B}^{i_{L}}=sin\left(i_{L}h_{res}\right)d_{range}\right\}\\ \; & I_{L}=\left\{i_{L}\;|\;i_{L}\;\in \;N,\;i_{L}<n_{L}\right\},\;n_{L}\;\in \;N \end{array}$$

where $x_{1B,2B}^{i_{L}}$ and $y_{1B,2B}^{i_{L}}$ represent the coordinates of each pair of points that form a beam and $n_{L}$ is the number of beams that compose $L_{B}$ ($n_{L}=h_{range}/h_{res}=900$).

In order to generate the distances measured by the sensor, in each sample time ($T_{m}^{L}=1/f_{m}^{L}$) the set $L_{B}$ is rotated and translated to Earth-axes as a function of the USV position vector, resulting in set $L_{E}$ [31]. Then, the points of intersection between the sets of segments $S_{E}$ and $L_{E}$ are calculated. Of all the points of intersection obtained for each beam, only the one closest to the USV is considered, while the rest of the intersections with $S_{E}$ are ignored. Thus, obstacles that are hidden due to the perspective effect are not detected. In addition, a measurement error is added to the detected points depending on their distance to the vehicle. As output, sensor model delivers a vector that contains the measured distances ($d_{m}^{i_{L}}$) at each angular position ($\theta_{m}^{i_{L}}$); see Equation (8). In this way, a difference with other works that also evaluate OA methods for USVs through numerical simulations [11,13,14,16,33,34,35,36,37,38,39,40,41,42,43], measurement uncertainty is added to the environmental information used by the RRSOAS.

(8) $$\begin{array}{c} \theta_{m}^{L}={\left(\begin{array}{ccccc} \theta_{m}^{0} & \cdots & \theta_{m}^{i_{L}} & \cdots & \theta_{m}^{n_{L}-1} \end{array}\right)}^{T},\;\theta_{m}^{i_{L}}=i_{L}h_{res}\\ D_{m}^{L}={\left(\begin{array}{ccccc} d_{m}^{0} & \cdots & d_{m}^{i_{L}} & \cdots & d_{m}^{n_{L}-1} \end{array}\right)}^{T},\;d_{m}^{i_{L}}=\left\{\begin{array}{cc} d_{min}^{i_{L}}+\eta^{i_{L}} & \mathrm{if}\;\;f_{Inter}^{i_{L}}=1\\ d_{range} & otherwise \end{array}\right. \end{array}$$

where $f_{Inter}^{i_{L}}=1$ represents that an intersection has occurred between the beam $\theta_{m}^{i_{L}}$ and some segment of $S_{E}$, $d_{min}^{i_{L}}$ is the minimum distance of each beam to the obstacles and $\eta^{i_{L}}$ is the WGN that affects each measurement, whose variance depends on $d_{min}^{i_{L}}$ [31].

### 2.3. Obstacle Scenarios Used to Evaluate the New RRSOAS

**Figure 3:**
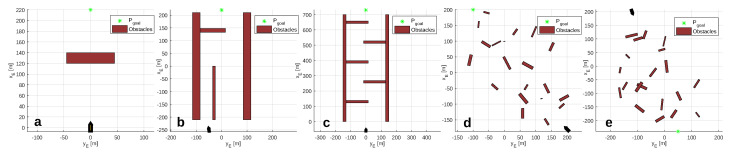
Obstacle scenarios used to evaluate the performances of SOA methods: (**a**) scenario 1, (**b**) scenario 2, (**c**) scenario 3, (**d**) scenario 4 and (**e**) scenario 5. Scenarios’ descriptions are available in [31].

This section presents the obstacle scenarios used to evaluate the RRSOAS. These scenarios have been established while taking into account the limitations of reactive obstacle avoidance methods [9,11,13,14,15,16,27,28,29,30,34,35,36,37,38,39,40,41,42,43,49,51], which are designed to avoid unknown obstacles that have not been considered in the path planning [1,2,3]. To this end, these methods use the environmental information processed in real time from the sensor measurements. Due to measurement errors and the limited ranges of these sensors, reactive methods do not guarantee that the vehicle will reach the goal if there are local minimums [12]. For this reason, in order to provide a global solution to the problem of autonomous navigation [1,2,3], reactive methods are combined with path planning algorithms [7,18,32,55]. Since in this work the obstacle scenarios are used to evaluate the RRSOAS, without considering its integration with any global algorithm, these do not contain local minimums (as might happen in some marine environments). Instead, unknown scenarios are used, which the USV detects during navigation through the measurements provided by the LIDAR model (7). Specifically, each obstacle scenario is defined by $S_{E}$ (6), the ocean current ($V_{c}$ and $\beta_{c}$), a goal waypoint ($P_{\mathrm{goal}}$), a goal velocity ($U_{goal}$, speed over ground [4]) and the initial vectors of position and velocity of the USV ($\eta_{0}$, $\nu_{0}$). These scenarios are used to study two features of the RRSOAS: performance and robustness. First, in order to evaluate the performance of the RRSOAS, it is compared with other reactive SOA methods. For this purpose, the five obstacle scenarios proposed in [31] and shown in Figure 3 are used.

However, there are infinite geometric/environmental combinations that define the possible scenarios where a USV can be located. Hence, once the OA system has been adjusted over specific scenarios, it is necessary to study its robustness in other scenarios whose obstacle distributions and environmental conditions are different. To this end, we evaluated the robustness of the RRSOAS through a statistical study. This study was based on the success rate of the reactive algorithm when it guided a USV over a population of randomized scenarios. With respect to most OA systems for USV [13,14,15,16,17,25,32,33,34,35,36,37,38,39,40,41,42,43,45,47] in which the results shown are limited to a number of specific scenarios, a more exhaustive robustness analysis was performed.

To carry out this study, a sample of scenarios ($n_{E}$) with a constant number of obstacles ($n_{o}$) was generated. These obstacles were randomly generated, as was the current direction, the initial position vector of the USV and the goal waypoint. In particular, the four points of the segments (6) were generated from a rectangle of random dimensions, position and orientation.

(9) $${\mathrm{Obst}}_{E}^{i_{O}}=\left(\begin{array}{cc} x_{1E}^{i_{O}} & y_{1E}^{i_{O}}\\ x_{2E}^{i_{O}} & y_{2E}^{i_{O}}\\ x_{3E}^{i_{O}} & y_{3E}^{i_{O}}\\ x_{4E}^{i_{O}} & y_{4E}^{i_{O}} \end{array}\right)=\underset{Rectangle\;Definition}{\underset{︸}{\frac{1}{2}\left(\begin{array}{cc} a^{i_{O}} & b^{i_{O}}\\ -a^{i_{O}} & b^{i_{O}}\\ -a^{i_{O}} & -b^{i_{O}}\\ a^{i_{O}} & -b^{i_{O}} \end{array}\right)\left(\begin{array}{cc} cos(\psi^{i_{O}}) & sin(\psi^{i_{O}})\\ -sin(\psi^{i_{O}}) & cos(\psi^{i_{O}}) \end{array}\right)}}+\underset{Rectangle\;Placement}{\underset{︸}{R^{i_{O}}\left(\begin{array}{c} 1\\ 1\\ 1\\ 1 \end{array}\right){\left(\begin{array}{c} cos(\theta^{i_{O}})\\ sin(\theta^{i_{O}}) \end{array}\right)}^{T}}}$$

Each random rectangle (${\mathrm{Obst}}_{E}^{i_{O}}$) is defined by its dimensions $\left(a^{i_{O}},b^{i_{O}}\right)$ and its orientation $\psi^{i_{O}}$, while its polar coordinates $\left(R^{i_{O}},\theta^{i_{O}}\right)$ place it on the scenario. These parameters are calculated as:

(10) $$a^{i_{O}}=a_{max}\zeta_{1}^{i_{O}},\;b^{i_{O}}=b_{max}\zeta_{2}^{i_{O}},\;\psi^{i_{O}}=\pi \zeta_{3}^{i_{O}},\;R^{i_{O}}=R_{E}\zeta_{4}^{i_{O}},\;\theta^{i_{O}}=\pi \left(2\zeta_{5}^{i_{O}}-1\right)$$

where $\zeta_{1\cdots 5}^{i_{O}}$ represents a uniformly distributed random number in the interval (0,1); $a_{max}$ and $b_{max}$ define the maximum dimensions of the rectangle; and finally, $R_{E}$ is a radius that adjusts the dispersion of the obstacles, which defines the occupation zone of the scenario.

As a starting point for all scenarios, the USV is outside of the occupation zone and its heading is pointed towards the goal waypoint, while sailing at a velocity $U_{goal}$. In this way, the reactive algorithm can always choose the most conservative solution: surround the scenario. In addition, the vessel is affected by a random direction current. Therefore, the initial position and velocity vectors of the USV, along with the $\beta_{c}$ angle, are defined as:

(11) $$\begin{array}{cc} \eta_{0}={\left(\begin{array}{ccc} x_{0} & y_{0} & \psi_{0} \end{array}\right)}^{T} & ={\left(\begin{array}{ccc} R_{0}cos\left(\theta_{0}\right) & R_{0}sin\left(\theta_{0}\right) & atan2(y_{0},\;x_{0})+\pi \end{array}\right)}^{T},\;\nu_{0}={\left(\begin{array}{ccc} U_{goal} & 0 & 0 \end{array}\right)}^{T}\\ \theta_{0}= & \pi \left(2\zeta_{\eta }-1\right),\;R_{0}=R_{E}+\frac{1}{2}\left(d_{range}+a_{max}\right),\;\beta_{c}=\pi \left(2\zeta_{\beta c}-1\right) \end{array}$$

where the variables $\left(\zeta_{\eta },\;\zeta_{\beta_{c}}\right)$ are random numbers uniformly distributed in the interval (0,1) and the polar coordinates $\left(R_{0},\;\theta_{0}\right)$ define the initial location of the USV, whose initial heading ($\psi_{0}$) is oriented towards the occupation zone.

On the other hand, the goal waypoint is located in an obstacle-free zone [12]. Moreover, since the USV begins pointing to $P_{\mathrm{goal}}$, it is defined as:

(12) $$P_{\mathrm{goal}}={\left(\begin{array}{cc} x_{goal} & y_{goal} \end{array}\right)}^{T}={\left(\begin{array}{cc} x_{0} & y_{0} \end{array}\right)}^{T}+2R_{0}{\left(\begin{array}{cc} cos(\psi_{0}) & sin(\psi_{0}) \end{array}\right)}^{T}$$

Finally, in line with most of the OA systems [11,12,13,14,16,17,26,27,30,33,40,41], it is necessary to define a goal course ($\chi_{goal}$) at each simulation instant (*k*). In this paper the USV’s mission is to arrive at $P_{\mathrm{goal}}$, so the goal course is defined by the Equation (13). If the mission involves following a concrete path, $\chi_{goal}$ must be calculated according to the kind of path [4,5,19,25].

(13) $$\chi_{goal}\left(k\right)=atan2\left(y_{goal}-y_{E}\left(k\right),\;x_{goal}-x_{E}\left(k\right)\right)+\psi \left(k\right)$$

**Figure 4:**
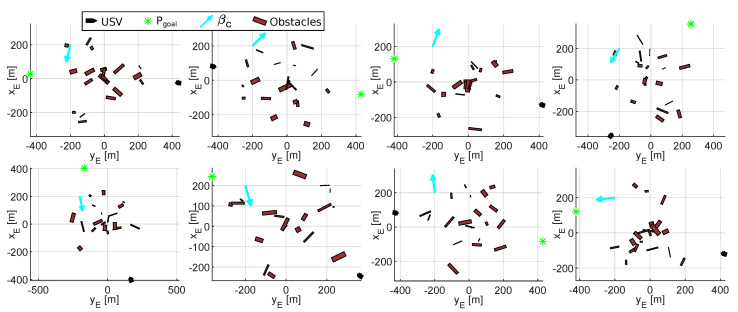
Random sample of obstacle scenarios, where $\Theta_{E}=\left(8\;20\;300\;75\;25\right)$.

In short, a sample of scenarios is defined by the vector $\Theta_{E}=\left(n_{E}\;n_{0}\;R_{E}\;a_{max}\;b_{max}\right)$, where $n_{E}$ is the number of scenarios that form the sample; $n_{0}$ sets the number of obstacles; $R_{E}$ limits the zone of occupation; and finally, $a_{max}$ and $b_{max}$ define the maximum dimensions of the obstacles. In addition, each sample of scenarios can be parameterized in terms of $U_{goal}$ and $V_{c}$. Thus, it is possible to study how the magnitudes of environmental disturbances affect the RRSOAS at different points of operation of the USV. As illustrative example, Figure 4 shows eight random scenarios generated by this procedure.

### 2.4. Model of the Environment Surrounding the USV: Occupation Grid

In order to generate a model of the environment that surrounds the USV, the obstacle detection systems [6,7,8,9,10] process and fusion the information obtained from different sensors and data sources (see Figure 1). Thus, the guidance system can use all the information to carry out a path planning and avoid any unforeseen obstacle. In this work, the obstacle detection system generates an Occupation Probability Grid (OPG) [6,8,29] which is used by the RRSOAS. OPGs allow decoupling the OA system and the complexity of the scenario (shape and number of obstacles). For this purpose, it is necessary to fix a resolution ($g_{res}$) in order to discretize the space around the vehicle in cells, which quantify the uncertainty contained in the measurements as an occupancy probability. Hence, the environmental analysis carried out by the reactive method depends only on the grid dimensions ($m_{g}\times n_{g}$). In this work, OPG is centered on the USV and it moves with the vehicle [29,47]. Specifically, the reference system of the OPG matches with the vessel’s local horizon reference system; see Figure 2. Furthermore, based on the numerical simulations carried out and in line with the cell size used in [47] for a vessel similar to the USV (1), the resolution has been set as $g_{res}=1$ m. Moreover, with the aim of using the maximum detection range of the sensor (7), the grid is set square and its dimension depends on $d_{range}$.

(14) $$i_{g}\in \left[1,n_{g}\right],\;j_{g}\in \left[1,m_{g}\right],\;n_{g}=m_{g}=2\left\lceil d_{range}/g_{res}\right\rceil +1$$

where ⌈x⌉ represents the function *floor(x)* and $\left(i_{g},\;j_{g}\right)$ are the indexes of the grid.

**Figure 5:**
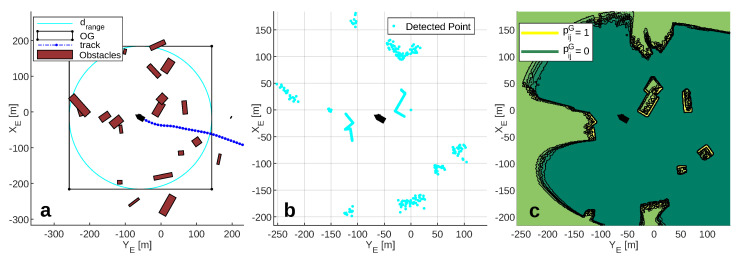
(**a**) Obstacle scenario. (**b**) LIDAR sensor measurements. (**c**) Occupancy probability grid.

Since this work is not focused on the development of a obstacle detection system, in the numerical simulations OPG was calculated using the *MATLAB Navigation Toolbox* [63]. In particular, the *occupancyMap* function was used to define OPG according to its dimensions and resolution. This function is based on a Bayesian filter, an approach widely used to generate OPGs [8]. Once the grid is defined, in each sample time $T_{m}^{L}$ the measurements delivered by the LIDAR sensor (7) are translated to this grid. For this purpose, the *insertRay* function is used, whose inputs are directly compatible with the measured distances (8) and the position vector of the USV, η(k). Furthermore, in order to avoid possible truncation errors caused by the discretization of the space, function *inflate* is used to expand each occupied cell a radius equivalent to $g_{res}$. Finally, the function *occupancyMatrix* is used to obtain the probability of occupation of all the cells ($p_{ij}$); see Equation (15). As a result, Figure 5 shows: the USV navigating over an obstacle scenario, the LIDAR sensor measurements and the OPG.

(15) $$g_{p}\left(k\right)=\left(\begin{array}{cccc} p_{11} & p_{12} & \cdots & p_{1m}\\ p_{21} & p_{22} & \cdots & p_{2m}\\ \vdots & \vdots & \ddots & \vdots \\ p_{n1} & p_{n2} & \cdots & p_{nm} \end{array}\right)=f_{B}\left(\theta_{m}^{L}\left(k\right),\;D_{m}^{L}\left(k\right),\;\eta \left(k\right),\;g_{p}\left(k-1\right)\right)$$

where $f_{B}$ represents the Bayesian filter used to generate the OPGs at each sample time *k*.

## 3. Robust Reactive Static Obstacle Avoidance System

**Figure 6:**
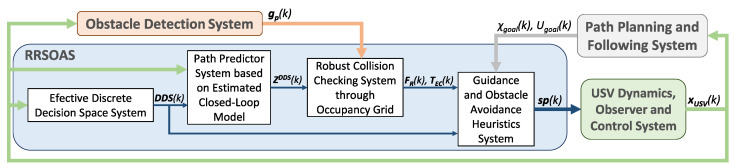
Functional diagram of the RRSOAS: inputs/outputs and the main subsystems.

This system has been designed from the synthesis of several basic concepts in the field of OA methods [12,27,29,30,51], taking into account general criteria used in USVs [16,17,34,40] and new contributions in this field in order to increase the robustness and applicability of SOA methods to vessels. A general scheme of this new method is shown in Figure 6. As inputs, the algorithm requires the state vector of the USV ($x_{\mathrm{USV}}$), an OPG ($g_{p}$) and goal setpoints ($\chi_{goal},\;U_{goal}$). As outputs, the RRSOAS generates the course and velocity setpoints $\mathrm{sp}\left(sp_{\chi },\;sp_{U}\right)$ for the USV controllers.

In each sample time ($T_{m}$) this reactive algorithm uses four subsystems:

- *Efective discrete decision space (DDS) System*. With the objective of avoiding a possible collision, this block generates a set of alternative government setpoints.
- *Path predictor system based on estimated closed-loop model*. Taking into account the USV’s state vector ($x_{\mathrm{USV}}$), DDS is used to make predictions of possible paths ($Z^{\mathrm{DDS}}$) that the USV could follow to avoid a collision.
- *Robust collision checking system through occupancy grid*. The $Z^{\mathrm{DDS}}$ paths are translated into the occupancy grid and characterized by a repulsive force ($F_{R}$) and an estimated collision time ($T_{\mathrm{EC}}$).
- *Guidance and obstacle avoidance heuristic system*. Finally, based on $F_{R}$ and $T_{\mathrm{EC}}$, and the goal setpoints ($\chi_{goal},\;U_{goal}$), a heuristic is used to restrict, weight and decide over DDS the setpoints that are demanded by the controllers; $\mathrm{sp}\left(sp_{\chi },\;sp_{U}\right)$.

### 3.1. Effective Discrete Decision Space System

When sailing in the presence of obstacles, a vessel’s current velocity and course can lead to a collision. In order to avoid such possible collisions, it is necessary to study a set of alternative setpoints for the course $\left(sp_{\chi }\right)$ and velocity $\left(sp_{U}\right)$ of the USV [11,12,13,14,16,17,41]. The combination of these alternative setpoints defines the decision space at each sample time (*k*):

(16) $$DS\left(k\right)=\left\{\mathrm{sp}\left(sp_{\chi },sp_{U}\right)\;|\;sp_{\chi }\in \left[\chi \left(k\right)-\Delta \chi_{max},\;\chi \left(k\right)+\Delta \chi_{max}\right],\;sp_{U}\in \left[0,\;U_{lim}\right]\right\}$$

where $\Delta \chi_{max}$ and $U_{lim}$ are adjustment parameters that define the maneuvering range of the USV, limiting the values of the alternative course/velocity setpoints.

Since the set (16) is continuous, it can be discretized according to a resolution [13,14,16,17,26,27,30,40,41]. A disadvantage when a fixed resolution is used for the course setpoints and DS is centered on the current vehicle course, a standard approach followed in mobile robotics [12,26,27,30,51], is that the resolution must be increased to reduce the tracking error of the goal course [40]. Consequently, the calculation time increases with the number of alternative setpoints. An alternative approach is to center DS directly on the goal course [16,37]. In this way, the authors ensure that the guidance of the USV follows $\chi_{goal}$ without the need to use high resolutions in the discretization of the course commands. Although, as a disadvantage, the symmetry (number of alternative setpoints to port and starboard) of space (16) depends on the angular distance between χ and $\chi_{goal}$. In order to reduce the tracking error with respect to $\chi_{goal}$ without using high resolutions, while DS is centered around χ, we propose an exponential discretization for the course setpoints; see Equation (17). This discretization limits candidate courses to bring navigation more in line with COLREGS [68], carrying out minor corrections to compensate any course changes due to disturbances and applying significant course changes (easily perceived by other vessels [68]) if there is a risk of collision. Moreover, although the obstacles present in the environment force the RRSOAS to demand changes of course in only one direction, the symmetry of space (16) is not affected.

(17) $$SP_{\chi }\left(k\right)=\left\{sp_{\chi }\;|\;sp_{\chi }=\chi \left(k\right)\pm \Delta \chi_{max}e^{\left(-i_{\chi }/\tau_{\chi }\right)},\;i_{\chi }\in \left[0,n_{\chi }\right]\right\}$$

where $\tau_{\chi }$ is a positive parameter that defines the variation of the resolution along the range $\Delta \chi_{max}$ and $n_{\chi }$ represents the even number of alternative courses (port/starboard symmetric).

On the other hand, the use of low resolutions to discretize velocity setpoints does not affect to the performances of OA systems for USVs [16,17]. For this reason, two fixed resolutions are used in this work: $U_{res}^{+}$ and $U_{res}^{-}$, which define alternative velocities higher and lower than $U_{goal}$, respectively. In addition, goal velocity and zero velocity (for unavoidable collision situations) are also considered as commands. Thus, the alternative setpoints for the velocity of navigation are given by:

(18) $$SP_{U}\left(k\right)=\left\{\begin{array}{cc} \left\{0,U_{goal}\left(k\right)\right\} & \mathrm{if}\;n_{U}=0\\ \left\{0,U_{goal}\left(k\right)\right\}\cup SP_{U}^{+}\cup SP_{U}^{-} & \mathrm{otherwise} \end{array}\right.$$

where $n_{U}$ is the even number of alternative velocities, while the sets $SP_{U}^{+}$ and $SP_{U}^{-}$ are defined as:

(19) $$\begin{array}{cc} SP_{U}^{+} & =\left\{sp_{U}\;|\;sp_{U}=U_{goal}\left(k\right)+i_{U}U_{res}^{+}\left(k\right)\right\},\;U_{res}^{+}\left(k\right)=\left(U_{lim}-U_{goal}\left(k\right)\right)/n_{U}\\ SP_{U}^{-} & =\left\{sp_{U}\;|\;sp_{U}=U_{goal}\left(k\right)+i_{U}U_{res}^{-}\left(k\right)\right\},\;U_{res}^{-}\left(k\right)=\left(U_{gov}-U_{goal}\left(k\right)\right)/n_{U} \end{array},\;i_{U}\in \left[1,n_{U}\right]$$

where $U_{gov}$ is the minimum velocity at which the course controller can govern the vessel.

**Figure 7:**
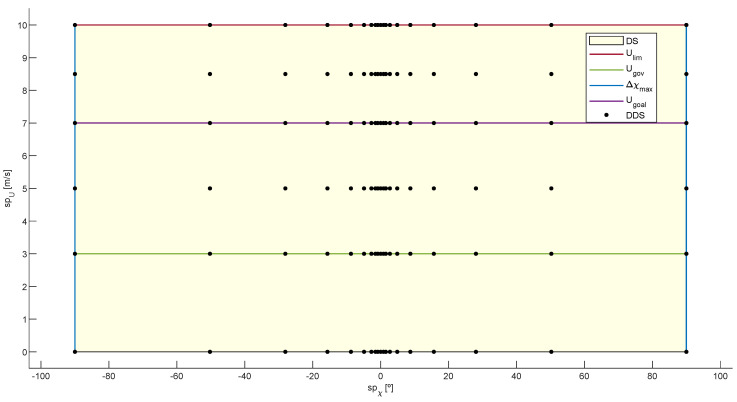
Discretization of space (16). Settings: $\chi \left(k\right)=0^{\circ }$, $n_{\chi }=9$, $\tau_{\chi }=1.7$ and $n_{U}=2$.

As a result, using an exponential resolution (17) to discretize the courses and two fixed resolutions (18) for the velocities, the decision space (16) is discretized according to Equation (20). Figure 7 shows one of the possible discrete decision space (DDS) that defines the set of alternative setpoints $\left(sp_{\chi },sp_{U}\right)$ evaluated by the RRSOAS.

(20) $$DDS\left(k\right)=\left\{{\mathrm{sp}}^{i_{c}}\left(sp_{\chi },sp_{U}\right)\;|\;sp_{\chi }\in SP_{\chi }\left(k\right),\;sp_{U}\in SP_{U}\left(k\right)\right\},\;i_{c}\in \left(\left[1,n_{c}\right]\cap N\right)$$

where ${\mathrm{sp}}^{i_{c}}\left(sp_{\chi },sp_{U}\right)$ represents a pair of possible government setpoints and $n_{c}=\left(2n_{\chi }+1\right)\left(2n_{U}+2\right)$ defines the total number of alternative government setpoints taken into account.

Finally, it is necessary to consider the oscillations that external disturbances (wind, waves and current) cause in the vessel’s course. These oscillations, although corrected by the autopilot in a finite time, may generate undesired changes in DDS (20), since these are not due to avoidance or following maneuvers. To reduce the variation of set (17) caused by these oscillations, in this work it is proposed to apply hysteresis (21) to the USV’s course, so χ is replaced by $\chi_{hyst}$ in Equations (16) and (17).

(21) $$\chi_{hyst}\left(k\right)=\left\{\begin{array}{cc} \chi (k) & \mathrm{if}\;|\chi \left(k\right)-\chi_{hyst}\left(k-1\right)|\geq \kappa_{\chi }\\ \chi_{hyst}\left(k-1\right) & \mathrm{otherwise} \end{array}\right.$$

where $\kappa_{\chi }$ is the hysteresis range, set according to the sea state and the autopilot’s performance.

### 3.2. Path Predictor System Based On Estimated Closed-Loop Model

This system estimates the possible future paths that the USV could take to avoid a collision. For this purpose, it does not need the mathematical modeling of the vessel or its controllers. Instead, it uses the new estimated closed-loop model proposed in this paper. Moreover, each prediction horizon is adjusted to ensure that all future paths associated with DDS (20) cover the same distance.

#### 3.2.1. Estimated Closed-Loop Model

In the first place, the closed-loop system formed by the vessel and the course/velocity controllers is modeled as two second-order differential equations with delay; see expressions (22) and (23). Each of these models relates the setpoints $\left(sp_{\chi },sp_{U}\right)$ with a linear estimation of the controlled variables $\left({\tilde{\chi }}_{l},{\tilde{U}}_{l}\right)$. Both models are defined by: effective time constants ($\tau_{\chi },\tau_{U}$), effective delay times ($d_{\chi },d_{U}$), damping coefficients ($\zeta_{\chi },\zeta_{U}$) and stationary gains ($K_{\chi },K_{U}$). These last ones, due to the error compensation made by the controllers to ensure that the USV follows the setpoints, are taken as $K_{\chi }=K_{U}=1$.

(22) $$\frac{d{\tilde{\chi }}_{l}\left(t\right)}{dt}={\tilde{r}}_{l}\left(t\right),\;\frac{d{\tilde{r}}_{l}\left(t\right)}{dt}=\frac{1}{\tau_{\chi }^{2}}\left(K_{\chi }sp_{\chi }\left(t-d_{\chi }\right)-2\zeta_{\chi }\tau_{\chi }{\tilde{r}}_{l}\left(t\right)-{\tilde{\chi }}_{l}\left(t\right)\right)$$

(23) $$\frac{d{\tilde{U}}_{l}\left(t\right)}{dt}={\tilde{a}}_{l}\left(t\right),\;\frac{d{\tilde{a}}_{l}\left(t\right)}{dt}=\frac{1}{\tau_{U}^{2}}\left(K_{U}sp_{U}\left(t-d_{U}\right)-2\zeta_{U}\tau_{U}{\tilde{a}}_{l}\left(t\right)-{\tilde{U}}_{l}\left(t\right)\right)$$

where ${\tilde{r}}_{l}$ and ${\tilde{a}}_{l}$ are estimates of the angular velocity and linear acceleration of the USV, respectively.

Taking into account the non-linear dynamics characteristic of marine surface vehicles and the strong coupling between their state variables [5,57,58,59,60], it is necessary to consider additional effects on the linear dynamics represented by models (22) and (23). First, speed falls due to changes of course are considered. Physically, this is caused by the coupling that exists between the components of the vessel’s velocity vector (ν) [5,57,58,59,60,69] and the drag forces generated by the action of the actuators; see Equations (1) and (3). To model this effect, the estimated linear velocity is redefined and depends on the estimated angular velocity:

(24) $$\tilde{U}\left(t\right)={\tilde{U}}_{l}\left(t-T_{p}\right)-\left|\tilde{r}\left(t-T_{p}\right)\right|\left(c_{1}{\tilde{U}}^{2}\left(t-T_{p}\right)+c_{2}\tilde{U}\left(t-T_{p}\right)+c_{3}\right),\;\tilde{U}\left(t\right)\in \left[U_{gov},\;U_{lim}\right]$$

where $\left(c_{1},c_{2},c_{3}\right)$ are positive coefficients that model the loss of velocity experienced by the vessel during a change of course, $T_{p}$ is the integration step and $\tilde{U}$ is limited to the USV navigation range.

Secondly, it is necessary to take into account the non-linear dynamics of the vehicle’s rotational rate. According to [5,57,58,59,60], in vessels the dynamics of *r* becomes faster when the forward velocity increases. Therefore, the parameters of the model (22) that define the rapidity of the course dynamics become non-linear and variable depending on $\tilde{U}$,

(25) $$\tau_{\chi }\left(t\right)=c_{4}+c_{5}/\tilde{U}\left(t-T_{p}\right)+c_{6}/{\tilde{U}}^{2}\left(t-T_{p}\right),\;d_{\chi }\left(t\right)=c_{7}+c_{8}/\tilde{U}\left(t-T_{p}\right)$$

where the coefficients $\left(c_{4},c_{5},c_{6},c_{7},c_{8}\right)$ are defined positive to ensure the stability of the ECLM and model how $\tau_{\chi }\left(t\right)$ and $d_{\chi }\left(t\right)$ decrease when the velocity of the vessel increases. Thus, the angular velocity and course of the new ECLM is defined as:

(26) $$\tilde{\chi }\left(t\right)=\int \tilde{r}\left(t\right)dt,\;\tilde{r}\left(t\right)=\int \frac{1}{\tau_{\chi }^{2}\left(t\right)}\left(sp_{\chi }\left(t-d_{\chi }\left(t\right)\right)-2\zeta_{\chi }\tau_{\chi }\left(t\right)\tilde{r}\left(t\right)-\tilde{\chi }\left(t\right)\right)dt,\;\tilde{r}\left(t\right)\in \left\{-r_{max},r_{max}\right\}$$

with $r_{max}$ as the maximum rotation rate of the vessel.

Finally, the positions $\left({\tilde{x}}_{E},{\tilde{y}}_{E}\right)$ are estimated according to:

(27) $${\tilde{x}}_{E}\left(t\right)=\int \tilde{U}\left(t\right)cos\left(\tilde{\chi }\left(t\right)\right)dt,\;{\tilde{y}}_{E}\left(t\right)=\int \tilde{U}\left(t\right)sin\left(\tilde{\chi }\left(t\right)\right)dt$$

Then, Equations (23)–(27) are discretized using the forward Euler method with the aim of predicting possible future paths. As a pre-tuning value, the integration step $T_{p}$ is established equal to the sample time of the Controllers (5). By regrouping terms, the new ECLM is obtained, a non-linear and time-varying model:

(28) $$\begin{array}{cc} \; & \underset{x_{\mathrm{ECLM}}\left(k\right)}{\underset{︸}{\left(\begin{array}{c} {\tilde{a}}_{l}\left(k\right)\\ {\tilde{U}}_{l}\left(k\right)\\ \tilde{U}\left(k\right)\\ \tilde{r}\left(k\right)\\ \tilde{\chi }\left(k\right)\\ {\tilde{x}}_{E}\left(k\right)\\ {\tilde{y}}_{E}\left(k\right) \end{array}\right)}}=\underset{A_{\mathrm{ECLM}}\left(k-1\right)}{\underset{︸}{\left(\begin{array}{ccccccc} a_{11} & a_{12} & 0 & 0 & 0 & 0 & 0\\ T_{p} & 1 & 0 & 0 & 0 & 0 & 0\\ 0 & 1 & a_{33} & 0 & 0 & 0 & 0\\ 0 & 0 & 0 & a_{44} & a_{45} & 0 & 0\\ 0 & 0 & 0 & T_{p} & 1 & 0 & 0\\ 0 & 0 & a_{63} & 0 & 0 & 1 & 0\\ 0 & 0 & a_{73} & 0 & 0 & 0 & 1 \end{array}\right)}}\underset{x_{\mathrm{ECLM}}\left(k-1\right)}{\underset{︸}{\left(\begin{array}{c} {\tilde{a}}_{l}\left(k-1\right)\\ {\tilde{U}}_{l}\left(k-1\right)\\ \tilde{U}\left(k-1\right)\\ \tilde{r}\left(k-1\right)\\ \tilde{\chi }\left(k-1\right)\\ {\tilde{x}}_{E}\left(k-1\right)\\ {\tilde{y}}_{E}\left(k-1\right) \end{array}\right)}}+\underset{B_{\mathrm{ECLM}}\left(k-1\right)}{\underset{︸}{\left(\begin{array}{cc} b_{11} & 0\\ 0 & 0\\ 0 & 0\\ 0 & b_{32}\\ 0 & 0\\ 0 & 0\\ 0 & 0 \end{array}\right)}}\underset{u_{\mathrm{ECLM}}\left(k-1\right)}{\underset{︸}{\left(\begin{array}{c} sp_{U}\left(k-\gamma_{1}\right)\\ sp_{\chi }\left(k-\gamma_{2}\right) \end{array}\right)}}\\ \; & \underset{y_{\mathrm{ECLM}}\left(k\right)}{\underset{︸}{\left(\begin{array}{c} \tilde{\chi }\left(k\right)\\ {\tilde{x}}_{E}\left(k\right)\\ {\tilde{y}}_{E}\left(k\right) \end{array}\right)}}=\underset{C_{\mathrm{ECLM}}}{\underset{︸}{\left(\begin{array}{ccccccc} 0 & 0 & 0 & 0 & 1 & 0 & 0\\ 0 & 0 & 0 & 0 & 0 & 1 & 0\\ 0 & 0 & 0 & 0 & 0 & 0 & 1 \end{array}\right)}}x_{\mathrm{ECLM}}\left(k\right)\;,\;\;\;\underset{Non-Time\;Dependent\;Parameters}{\underset{︸}{\left(\begin{array}{c} a_{11}\\ a_{12}\\ \gamma_{1} \end{array}\right)=\left(\begin{array}{c} 1-\left(2\zeta_{U}T_{p}\right)/\tau_{U}\\ -T_{p}/\tau_{U}^{2}\\ 1+\lceil d_{U}/T_{p}\rceil \end{array}\right)}}\\ \; & \underset{Time\;Dependent\;Parameters\;(k-1)}{\underset{︸}{\left(\begin{array}{c} a_{33}\\ a_{44}\\ a_{45}\\ a_{63}\\ a_{73}\\ \gamma_{2} \end{array}\right)=\left(\begin{array}{cccccc} c_{1} & c_{3} & 0 & 0 & 0 & 0\\ 0 & 0 & 2\zeta_{\chi } & 0 & 0 & 0\\ 0 & 0 & 1/\tau_{\chi } & 0 & 0 & 0\\ 0 & 0 & 0 & 1 & 0 & 0\\ 0 & 0 & 0 & 0 & 1 & 0\\ 0 & 0 & 0 & 0 & 0 & 1 \end{array}\right)\left(\begin{array}{c} -|\tilde{r}|\tilde{U}\\ -|\tilde{r}|/\tilde{U}\\ -T_{p}/\tau_{\chi }\\ T_{p}cos\left(\tilde{\chi }\right)\\ T_{p}sin\left(\tilde{\chi }\right)\\ \left\lceil d_{\chi }/T_{p}\right\rceil \end{array}\right)-\left(\begin{array}{c} |\tilde{r}|c_{2}\\ -1\\ 0\\ 0\\ 0\\ -1 \end{array}\right),\;\left(\begin{array}{c} \tau_{\chi }\\ d_{\chi } \end{array}\right)=\left(\begin{array}{ccc} c_{4} & c_{5} & c_{6}\\ c_{7} & c_{8} & 0 \end{array}\right)\left(\begin{array}{c} 1\\ 1/\tilde{U}\\ 1/{\tilde{U}}^{2} \end{array}\right)}} \end{array}$$

where ⌈x⌉ represents the function *floor(x)*, $b_{32}=-a_{45}$ and $b_{11}=-a_{12}$.

As can be seen, the dynamics of the ECLM is defined by the parameter vector (29). To identify this vector, in a real vehicle, time series would be recorded in which an experienced captain performs obstacle avoidance maneuvers with the USV (logging the vessel’s course, linear and angular velocities). Thus, the ECLM takes into account the dynamics of the USV during collision risk situations.

(29) $$\Theta_{\mathrm{ECLM}}=\left(\begin{array}{cccccccccccc} \tau_{U} & d_{U} & \zeta_{U} & \zeta_{\chi } & c_{1} & c_{2} & c_{3} & c_{4} & c_{5} & c_{6} & c_{7} & c_{8} \end{array}\right)$$

Since we do not have a real vessel, the RRSOAS algorithm itself has been used as an experienced pattern to generate the time series that will be used in order to identify the model (28). In these simulations, the predictions used by the RRSOAS are made from the models (1), (3) and (5), which have been discretized with the forward Euler method and an integration step $T_{p}=0.1$ s.

Moreover, as the ECLM is a system with delays, in line with the work carried out in [70,71,72], a numerical optimization method based on a genetic algorithm (GA, [73]) is used for the identification of the vector (29). In this way, each $\Theta_{\mathrm{ECLM}}$ is considered as an individual of a population, and consequently, each parameter represents a gene. In addition, to quantify the fit of the model (28) to the time series, the SAE (sum absolute errors) indicators are used:

(30) $$SAE_{\chi }=\sum_{k=1}^{n_{ts}}\left|\chi \left(k\right)-\tilde{\chi }\left(k\right)\right|,\;SAE_{r}=\sum_{k=1}^{n_{ts}}\left|r\left(k\right)-\tilde{r}\left(k\right)\right|,\;SAE_{U}=\sum_{k=1}^{n_{ts}}\left|U\left(k\right)-\tilde{U}\left(k\right)\right|$$

where $n_{ts}=T_{t}/T_{p}$ is an integer that represents the length of the time series, which are obtained in navigation trials, and $T_{t}$ is its duration.

Accordingly, the cost function used by the GA to calculate the fit of each individual is defined as:

(31) $$J_{ECLM}=SAE_{\chi }/\pi +SAE_{r}/r_{max}+SAE_{U}/U_{lim}$$

**Figure 8:**
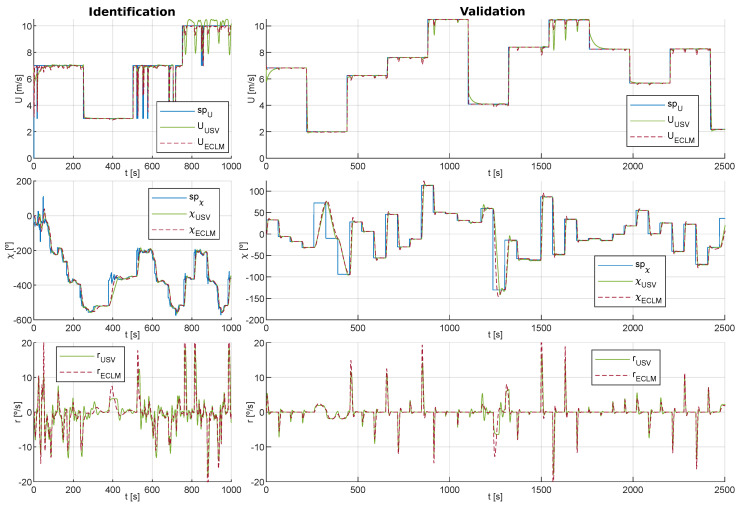
Time series obtained through numerical simulations with the unmanned surface vehicle (USV), Equations (1), (3) and (5), versus ECLM (28). Two time series: identification and validation.

Due to the random nature of evolutionary algorithms [73], the identification for the same time series has been repeated 32 times, and from the parameter vectors obtained, the one that best fits a second validation time series was chosen; see Figure 8.

To implement the GA, Toolbox within Matlab was used [74]. In order to ensure the stability of the model and to limit the search space, the parameters of the vector $\Theta_{\mathrm{ECLM}}$ were restricted while taking into account its physical meaning and the USV’s behavior. Consequently, the option *mutationadaptfeasible* was set as a mutation function [74] and the initial population was also limited. In addition, we allowed: a population of 200 individuals, a maximum of 100 generations and the parameter *MaxStallGenerations* was established as 15% of the maximum of generations. Finally, to avoid early convergence [73,74], elite individuals were limited to 2% of the population and the crossover factor was reduced (increasing the mutation factor) to 0.7. The other parameters were the ones established by default in [74]. As a result, the parameters identified for the ECLM, which estimates the dynamics of the closed-loop system formed by Equations (1), (3) and (5), were as follows:

(32) $${\tilde{\Theta }}_{\mathrm{ECLM}}=\left(\begin{array}{cccccccccccc} 0.7 & 0.2 & 0.9 & 0.6 & 0.0002 & 0.0003 & 0.015 & 0.1 & 15.6 & 49 & 0.8 & 5.7 \end{array}\right)$$

#### 3.2.2. Path Predictor with Variable Prediction Horizon

The path predictor proposed in this work uses a variable prediction horizon $M^{i_{c}}$ to ensure that all future paths cover the same prediction distance ($D_{p}$). In this way, the prediction time is adapted to each pair of ${\mathrm{sp}}^{i_{c}}$ setpoints as a function of USV’s dynamics. In particular, we propose to set $D_{p}$ according to the measurement range of the sensors ($d_{range}$). Furthermore, if this measurement range is oversized due to the maneuverability of the USV to avoid static obstacles, the distance $D_{p}$ can be reduced as long as it ensures that the course and speed of the ECLM (28) reaches the stationary regime.

From the current state of the vessel, the ECLM status vector is obtained to initialize the predictions:

(33) $$x_{\mathrm{ECLM}}\left(k\right)={\left(\begin{array}{ccccccc} {\tilde{a}}_{l}\left(k\right) & {\tilde{U}}_{l}\left(k\right) & U(k) & r(k) & \chi (k) & x_{E}\left(k\right) & y_{E}\left(k\right) \end{array}\right)}^{T}$$

where ${\tilde{U}}_{l}\left(k\right)$ is obtained according to the Equation (24) and ${\tilde{a}}_{l}\left(k\right)$ is calculated using Euler’s forward approximation:

(34) $${\tilde{U}}_{l}\left(k\right)=U\left(k\right)+\left|r(k)\right|\left(c_{1}U^{2}\left(k\right)+c_{2}U\left(k\right)+c_{3}\right),\;{\tilde{a}}_{l}\left(k\right)=\frac{U(k)-U(k-1)}{T_{m}}$$

In addition, due to the delays defined in the ECLM (28), the control setpoints demanded by the controllers in previous periods are recorded:

(35) $$\begin{array}{cc} \; & {\mathrm{sp}}_{\chi }^{\mathrm{past}}=\left(\begin{array}{ccc} sp_{\chi }\left(k-1\right) & \cdots & sp_{\chi }\left(k-\gamma_{2}^{b}\right) \end{array}\right)\\ \; & {\mathrm{sp}}_{U}^{\mathrm{past}}=\left(\begin{array}{ccc} sp_{U}\left(k-1\right) & \cdots & sp_{U}\left(k-\gamma_{1}\right) \end{array}\right) \end{array}\;,\;\gamma_{2}^{b}=\left\lceil \frac{c7+c_{8}/U_{gov}}{T_{p}}\right\rceil +1$$

where $\gamma_{2}^{b}$ is the maximum delay taken into account for the estimation of course dynamics. This delay is obtained at the minimum government velocity $U_{gov}$; see Equations (24) and (25).

With the initial state (33) and previous ECLM entries (35) established, each pair of alternative government setpoints is held for the entire prediction horizon. As a result, by carrying out iterative calculations with the ECLM (28), an estimated future path is obtained for each pair of ${\mathrm{sp}}^{i_{c}}$,

(36) $$Y^{i_{c}}\left(k\right)=\left(\begin{array}{ccccc} y_{\mathrm{ECLM}}\left(k+1\right) & \ldots & y_{\mathrm{ECLM}}\left(k+m^{i_{c}}\right) & \ldots & y_{\mathrm{ECLM}}\left(k+M^{i_{c}}\right) \end{array}\right),\;m^{i_{c}}\in \left[1,\;M^{i_{c}}\right]$$

According to Equation (37), each horizon $M^{i_{c}}$ must ensure that the path reaches the prediction distance $D_{p}$, which has been set equal to the measurement range of the LIDAR sensor (7). As an exception, if a zero velocity setpoint is evaluated, $M^{i_{c}}$ is truncated when $U_{gov}$ is reached. In a real hazardous situation, when a stop command is demanded, it will be necessary to switch the USV to dynamic positioning mode, or if it is not available, manual/emergency mode.

(37) $$M^{i_{c}}=\left\{\begin{array}{cc} m^{i_{c}} & \mathrm{if}\;\tilde{d}\left(k+m^{i_{c}}\right)\geq D_{p}\\ M_{max} & \mathrm{otherwise} \end{array}\right.$$

where $\tilde{d}\left(k+m^{i_{c}}\right)$ is the distance traveled in each prediction step and $M_{max}$ represents the maximum prediction horizon allowed.

With the aim of characterizing each possible future path, the distance traveled $\tilde{d}\left(k+m^{i_{c}}\right)$ and the prediction time $\tilde{t}\left(k+m^{i_{c}}\right)$ are calculated. For that purpose, the vector (36) was extended:

(38) $$Z^{i_{c}}\left(k\right)=\left(\begin{array}{c} Y^{i_{c}}\left(k\right)\\ D^{i_{c}}\left(k\right)\\ T^{i_{c}}\left(k\right) \end{array}\right),\;\begin{array}{cc} \; & D^{i_{c}}\left(k\right)=\left(\begin{array}{ccccc} \tilde{d}\left(k+1\right) & \ldots & \tilde{d}\left(k+m^{i_{c}}\right) & \ldots & \tilde{d}\left(k+M^{i_{c}}\right) \end{array}\right)\\ \; & T^{i_{c}}\left(k\right)=\left(\begin{array}{ccccc} \tilde{t}\left(k+1\right) & \ldots & \tilde{t}\left(k+m^{i_{c}}\right) & \ldots & \tilde{t}\left(k+M^{i_{c}}\right) \end{array}\right) \end{array}$$

By grouping the vectors $Z^{i_{c}}$, the possible future paths for the entire DDS (20) are obtained:

(39) $$Z^{\mathrm{DDS}}\left(k\right)=\left(\begin{array}{ccccc} Z^{1}\left(k\right) & \cdots & Z^{i_{c}}\left(k\right) & \cdots & Z^{n_{c}}\left(k\right) \end{array}\right)$$

### 3.3. Robust Collision Checking System through Occupancy Grid

In this work, two novel contributions have been developed to increase the robustness of OA methods applied to USVs [16,17,34,36,39,40,44,46]. The first one takes into account the uncertainty present in the mathematical modeling of the USV that is used to make predictions. The second contribution deals with the measurement/estimation errors contained in the environmental model.

#### 3.3.1. Variable Shape as a Function of the Prediction Step

Due to the recursive character of the predictions, parametric and structural uncertainties present in mathematical modeling [66] cause prediction errors that increase with each step ($m^{i_{c}}$). In order to take into account these errors, as well as the shape of the vessel, we propose to model the USV’s contour as an ellipse whose perimeter varies as a function of $m^{i_{c}}$. For this purpose, the shape of the vessel is approximated to a discrete ellipse referenced to body-axis system; see Equation (40). In this ellipse, the major and minor diagonals are sized according to the length and the beam of the USV ($L_{USV}$ and $M_{USV}$). On the other hand, the number of points used to discretize the ellipse, $n_{\epsilon }$, can be calculated according to an approximation of its perimeter (*Ramanujan II-Cantrell*, [75]) and the resolution of the occupation grid ($g_{res}$). However, with the aim of reducing the algorithm’s run time, $n_{\epsilon }$ can be tuned based on the computational capacity of the hardware employed.

(40) $$\epsilon_{B}=\left(\begin{array}{cc} x_{\epsilon B}^{1} & y_{\epsilon B}^{1}\\ \vdots & \vdots \\ x_{\epsilon B}^{i_{\epsilon }} & y_{\epsilon B}^{i_{\epsilon }}\\ \vdots & \vdots \\ x_{\epsilon B}^{n_{\epsilon }} & y_{\epsilon B}^{n_{\epsilon }} \end{array}\right)=\frac{1}{2}\left(\begin{array}{cc} cos\left(res_{\epsilon }\right) & sin\left(res_{\epsilon }\right)\\ \vdots & \vdots \\ cos\left(i_{\epsilon }res_{\epsilon }\right) & sin\left(i_{\epsilon }res_{\epsilon }\right)\\ \vdots & \vdots \\ cos\left(n_{\epsilon }res_{\epsilon }\right) & sin\left(n_{\epsilon }res_{\epsilon }\right) \end{array}\right)\left(\begin{array}{cc} \gamma_{L}L_{USV} & 0\\ 0 & \gamma_{M}M_{USV} \end{array}\right),\;\begin{array}{cc} \; & i_{\epsilon }\in \left[1,n_{\epsilon }\right]\\ \; & res_{\epsilon }=2\pi /n_{\epsilon } \end{array}$$

where $\gamma_{L}$ and $\gamma_{M}$ are safety factors used to oversize the vessel’s shape.

The points $\epsilon_{B}$ are translated to the reference system of the occupancy grid or local-axis system,

(41) $$\epsilon_{L}\left(k+m^{i_{c}}\right)=\epsilon_{B}\left(\begin{array}{cc} \;\;\;cos\left(\tilde{\chi }\left(k+m^{i_{c}}\right)\right) & sin\left(\tilde{\chi }\left(k+m^{i_{c}}\right)\right)\\ -sin\left(\tilde{\chi }\left(k+m^{i_{c}}\right)\right) & cos\left(\tilde{\chi }\left(k+m^{i_{c}}\right)\right) \end{array}\right)+M_{\mathrm{ones}}d_{L}\left(k+m^{i_{c}}\right)$$

where $M_{\mathrm{ones}}$ represents a unit matrix of dimension $n_{\epsilon }\times 2$, while $d_{L}$ is defined as:

(42) $$d_{L}\left(k+m^{i_{c}}\right)=\mathrm{diag}\left({\tilde{x}}_{E}\left(k+m^{i_{c}}\right)-x_{E}\left(k\right),\;\;{\tilde{y}}_{E}\left(k+m^{i_{c}}\right)-y_{E}\left(k\right)\right)$$

**Figure 9:**
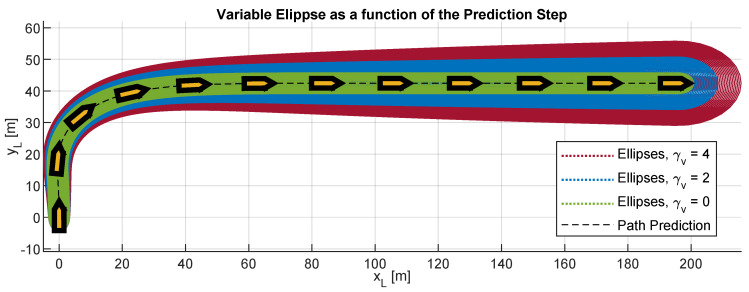
Variable ellipses obtained for a prediction horizon $M^{i_{c}}$; $n_{\epsilon }=32$ has been set.

To consider the prediction errors due to the uncertainty present in the model, the constant dimension ellipse used in Equation (41) is replaced by an ellipse whose perimeter increases with $m^{i_{c}}$; see Equation (43). Moreover, to model and limit the growth of this ellipse, it is proposed to use a hyperbolic tangent (function with continuous bounds). As a visual example, Figure 9 shows a comparative of the ellipses $\epsilon_{B}^{v}$ obtained when $\gamma_{v}$ is modified.

(43) $$\epsilon_{B}^{v}\left(k+m^{i_{c}}\right)=\epsilon_{B}\left(1+tanh\left(\frac{m^{i_{c}}}{M^{i_{c}}}\right)\gamma_{v}\right)$$

where $\gamma_{v}$ is defined positive and represents a variable oversize factor, which is established according to the degree of fit between the prediction model and the real behavior of the USV.

#### 3.3.2. New Repulsive Forces and Estimated Collision Times Method

In order to translate the possible future paths to the occupancy probability grid ($g_{p}$), at each prediction step, the points of the discrete ellipse (41) are expressed as cells:

(44) $$\epsilon_{\mathrm{ij}}\left(k+m^{i_{c}}\right)={\left(\begin{array}{ccccc} i_{g}^{1} & \cdots & i_{g}^{i_{\epsilon }} & \cdots & i_{g}^{n_{\epsilon }}\\ j_{g}^{1} & \cdots & j_{g}^{i_{\epsilon }} & \cdots & j_{g}^{n_{\epsilon }} \end{array}\right)}^{T}=\left\lfloor \epsilon_{L}\left(k+m^{i_{c}}\right)\left(\begin{array}{cc} -1/g_{res} & 0\\ 0 & 1/g_{res} \end{array}\right)\right\rceil +b_{g}M_{\mathrm{ones}}$$

where ⌊x⌉ represents the function *round(x)* and $b_{g}=\left(1+n_{g}\right)/2$.

From the probability of occupation stored in the cells of $\epsilon_{\mathrm{ij}}$ and $p_{\epsilon }$, the maximum probability is obtained for each step of the prediction ($p_{max}$); see Equation (45). Then, by grouping $p_{max}\left(k+m^{i_{c}}\right)$ for the entire prediction horizon, the vector that contains the maximum occupancy probabilities is formed for each possible future path; see Equation (46).

(45) $$p_{max}\left(k+m^{i_{c}}\right)=max\left(p_{\epsilon }\left(k+m^{i_{c}}\right)\right),\;p_{\epsilon }\left(k+m^{i_{c}}\right)=\left(\begin{array}{ccccc} p_{ij}^{1} & \cdots & p_{ij}^{i_{\epsilon }} & \cdots & p_{ij}^{n_{\epsilon }} \end{array}\right)$$

(46) $$P_{\max}^{i_{c}}\left(k\right)=\left(\begin{array}{ccccc} p_{max}\left(k+1\right) & \cdots & p_{max}\left(k+m^{i_{c}}\right) & \cdots & p_{max}\left(k+M^{i_{c}}\right) \end{array}\right)$$

**Figure 10:**
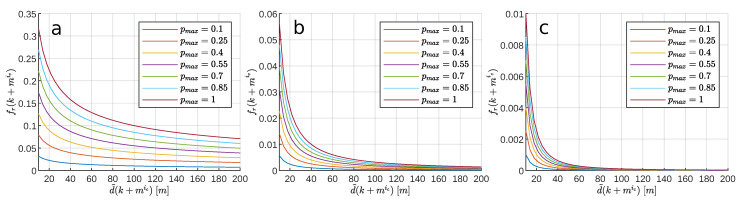
Repulsive forces as a function of possible future distance, maximum probability of occupancy and different values of $n_{d}$; (**a**) $n_{d}=0.5$, (**b**) $n_{d}=1.25$ and (**c**) $n_{d}=2$.

Now, the concept of repulsive forces ($f_{r}$) from the VFF (virtual force field, [29]) method is used. In this method, all cells of the grid produce a $f_{r}$ depending on their current distance to the vehicle and their probability of occupancy. Then, these repulsive forces are added, together with a attraction force, to obtain a resultant guidance force. As a novel contribution, in this work the repulsive forces (47) are used to characterize each possible future path $Z^{i_{c}}$. For this purpose, at each prediction step $m^{i_{c}}$, the force $f_{r}\left(k+m^{i_{c}}\right)$ is calculated as a function of the future distance $\tilde{d}\left(k+m^{i_{c}}\right)$ and the maximum probability of the cells crossed by the discrete ellipse (44); $p_{max}\left(k+m^{i_{c}}\right)$.

(47) $$f_{r}^{i_{c}}\left(k\right)=\left(\begin{array}{ccccc} f_{r}\left(k+1\right) & \cdots & f_{r}\left(k+m^{i_{c}}\right) & \cdots & f_{r}\left(k+M^{i_{c}}\right) \end{array}\right),\;f_{r}\left(k+m^{i_{c}}\right)=\frac{p_{max}\left(k+m^{i_{c}}\right)}{{\tilde{d}}^{n_{d}}\left(k+m^{i_{c}}\right)}$$

where $n_{d}$ defines the variation of the repulsive force as a function of the future distance. In the VFF method [29], the authors set $n_{d}=2$. Specifically, they carry out the autonomous guidance of the CARMEL mobile robot, which is equipped with 24 ultrasounds whose detection range covers 3 m. Since this measurement range is much lower than the one used in USVs [1,2,3,13,31,41,67], it is necessary to study the variation of repulsive forces as a function of the parameter $n_{d}$. To this end, Figure 10 shows the repulsive forces obtained, over the measurement range used in this work ($d_{range}=200$ m), for three different values of $n_{d}$. Due to the variation shown in this figure, and with the reason that all obstacles located within $d_{range}$ produce a significant repulsive force, in this work $n_{d}=0.5$ has been set.

On the other hand, in order to constrain the setpoints ${\mathrm{sp}}^{i_{c}}$ that would lead to an immediate collision, we propose to estimate the collision times for each possible future path; $T_{ec}^{i_{c}}$. For this purpose, at each prediction step $m^{i_{c}}$, $t_{ec}\left(k+m^{i_{c}}\right)$ is calculated as a function of the prediction time $\tilde{t}\left(k+m^{i_{c}}\right)$ and the maximum probability $p_{max}\left(k+m^{i_{c}}\right)$; see Equation (48). Then, by grouping $t_{ec}\left(k+m^{i_{c}}\right)$ for the entire prediction horizon $M^{i_{c}}$, the vector (49) is formed.

(48) $$t_{ec}\left(k+m^{i_{c}}\right)=\left\{\begin{array}{cc} \frac{\tilde{t}\left(k+m^{i_{c}}\right)}{p_{max}^{n_{t}}\left(k+m^{i_{c}}\right)} & \mathrm{if}\;p_{max}\left(k+m^{i_{c}}\right)>0\\ M_{max}T_{P} & otherwise \end{array}\right.,\;n_{t}\in \left(0,1\right]$$

where $n_{t}$ is an parameter used to oversize the occupancy probability, and consequently, decrease the estimated collision time.

(49) $$t_{\mathrm{ec}}^{i_{c}}\left(k\right)=\left(\begin{array}{ccccc} t_{ec}\left(k+1\right) & \cdots & t_{ec}\left(k+m^{i_{c}}\right) & \cdots & t_{ec}\left(k+M^{i_{c}}\right) \end{array}\right)$$

Finally, looking for the worst case, each ${\mathrm{sp}}^{i_{c}}$ is defined by the highest repulsive force ($F_{R}^{i_{c}}$) and the lowest estimated collision time ($T_{EC}^{i_{c}}$). Thus, once all alternative government setpoints have been evaluated, the DDS (20) is characterized by the prediction model and the occupancy probability grid:

(50) $$\begin{array}{cc} \; & F_{R}^{\mathrm{DDS}}\left(k\right)=\left(\begin{array}{ccccc} F_{R}^{1}\left(k\right) & \cdots & F_{R}^{i_{c}}\left(k\right) & \cdots & F_{R}^{n_{c}}\left(k\right) \end{array}\right),\;\;\;F_{R}^{i_{c}}\left(k\right)=max\left(f_{r}^{i_{c}}\left(k\right)\right)\\ \; & T_{\mathrm{EC}}^{\mathrm{DDS}}\left(k\right)=\left(\begin{array}{ccccc} T_{EC}^{1}\left(k\right) & \cdots & T_{EC}^{i_{c}}\left(k\right) & \cdots & T_{EC}^{n_{c}}\left(k\right) \end{array}\right),\;T_{EC}^{i_{c}}\left(k\right)=min\left(t_{\mathrm{ec}}^{i_{c}}\left(k\right)\right) \end{array}$$

### 3.4. Guidance and Obstacle Avoidance Heuristic System

In line with most reactive obstacles avoidance methods [12], a heuristic is minimized to decide the setpoints $\mathrm{sp}(sp_{\chi },sp_{U})$ demanded by the controllers at each sample time (*k*). In particular, this heuristic is a modification of the one proposed for the dynamic windows method [27], with the purpose of improving its performance in USVs.

(51) $$\begin{array}{cc} \; & J_{\mathrm{RRSOAS}}\left(k\right)=\alpha_{1}J_{\mathrm{Heading}}\left(k\right)+\alpha_{2}J_{\mathrm{Velocity}}\left(k\right)+\alpha_{3}J_{\mathrm{FR}}\left(k\right)+\alpha_{4}J_{\mathrm{Past}}\left(k\right)\\ \; & T_{mac}M_{\mathrm{ones}}\leq T_{\mathrm{EC}}^{\mathrm{DDS}}\left(k\right) \end{array}$$

where $\alpha_{i}$, for i∈{1,2,3,4}, represent the tuning parameters used in the heuristic weighting, $M_{\mathrm{ones}}$ is a unit matrix of dimension $1\times n_{c}$, and as in [27], $T_{mac}$ is a parameter used to restrict the setpoints ${\mathrm{sp}}^{i_{c}}$ according to their estimated collision times. In addition, in order to facilitate the tuning of the algorithm, the indeces taken into account in the heuristics are normalized.

First, based on the proposal made in [27], the indices $J_{\mathrm{Heading}}$ and $J_{\mathrm{Velocity}}$ are obtained, which define the alignment of the DDS (20) with the goal course and velocity. Moreover, in line with the proposal initially made in [30], and later, used in other OA systems for USVs [16,17], the index $J_{\mathrm{Past}}$ is added to take into account the alignment of each $sp_{\chi }^{i_{c}}\left(k\right)$ with the previous course setpoints $sp_{\chi }^{i_{c}}\left(k-1\right)$. In this way, the algorithm has a memory effect that reduces commutations in the course setpoints. These commutations, due to small variations in the heuristics between different execution periods, cause indecisive behavior in the USV’s guidance that can lead to a collision [30]. In particular, by expressing sets (17) and (18) in vector form:

(52) $$\begin{array}{cc} \; & {\mathrm{SP}}_{\chi }^{\mathrm{DDS}}\left(k\right)=\left(\begin{array}{ccccc} sp_{\chi }^{1}\left(k\right) & \cdots & sp_{\chi }^{i_{c}}\left(k\right) & \cdots & sp_{\chi }^{n_{c}}\left(k\right) \end{array}\right)\\ \; & {\mathrm{SP}}_{U}^{\mathrm{DDS}}\left(k\right)=\left(\begin{array}{ccccc} sp_{U}^{1}\left(k\right) & \cdots & sp_{U}^{i_{c}}\left(k\right) & \cdots & sp_{U}^{n_{c}}\left(k\right) \end{array}\right) \end{array}$$

From the vectors (52), the previous course setpoint $sp_{\chi }\left(k-1\right)$ and the goal setpoints $\left(\chi_{goal},U_{goal}\right)$, the indices $J_{Heading}$, $J_{Past}$ and $J_{Velocity}$ are obtained:

(53) $$\begin{array}{cc} \; & J_{\mathrm{Heading}}\left(k\right)=\frac{1}{\pi }\Delta \left({\mathrm{SP}}_{\chi }^{\mathrm{DDS}}\left(k\right),\;\chi_{goal}\left(k\right)M_{\mathrm{ones}}\right),\;J_{\mathrm{Past}}\left(k\right)=\frac{1}{\pi }\Delta \left({\mathrm{SP}}_{\chi }^{\mathrm{DDS}}\left(k\right),\;sp_{\chi }\left(k-1\right)M_{\mathrm{ones}}\right)\\ \; & J_{\mathrm{Velocity}}\left(k\right)=\frac{1}{\Upsilon_{U}^{max}\left(k\right)}\Upsilon_{U}\left(k\right),\;\Upsilon_{U}^{max}\left(k\right)=max\left(\Upsilon_{U}\left(k\right)\right),\;\Upsilon_{U}\left(k\right)=|{\mathrm{SP}}_{U}^{\mathrm{DDS}}\left(k\right)-U_{goal}\left(k\right)M_{\mathrm{ones}}| \end{array}$$

where Δ and || calculate the absolute angular distance and the absolute value, respectively.

On the other hand, instead of weighting the collision distances [16,17,27,40], in this work the index $J_{\mathrm{FR}}$ (54) is employed. Thus, when weighting the DDS (20), the occupancy probability of the space is considered through the repulsive forces that characterize each possible future path.

(54) $$J_{\mathrm{FR}}\left(k\right)=\frac{1}{F_{R}^{max}\left(k\right)}F_{R}^{\mathrm{DDS}}\left(k\right),\;F_{R}^{max}\left(k\right)=max\left(F_{R}^{\mathrm{DDS}}\left(k\right)\right)$$

Finally, if the DDS (20) is empty, which means that all alternative government setpoints have been restricted by the heuristics (51), the RRSOAS will demand a stop command. Consequently, the USV will switch to manual/emergency mode or, if a dynamic positioning control is available [4,5], the yaw of the vessel could be modified to restart the mission in a safe manner.

## 4. Simulation Results

**Figure 11:**
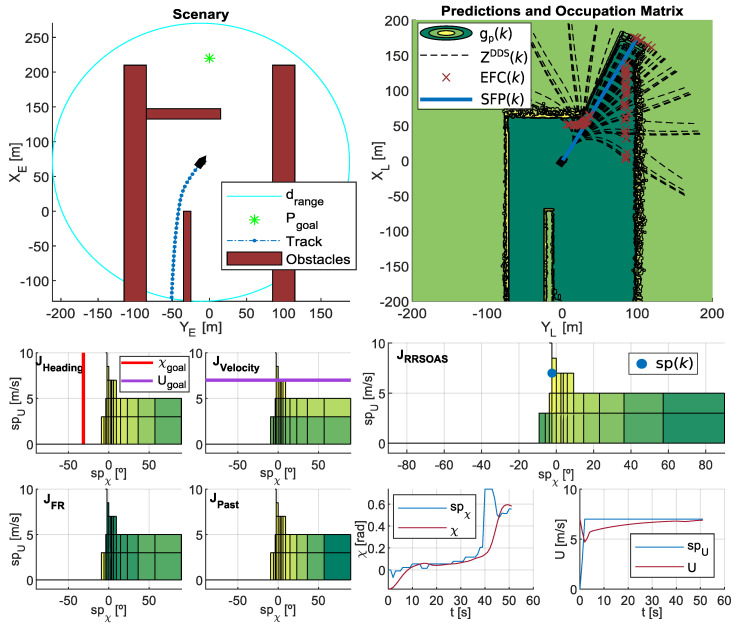
A RRSOAS execution period: evolution of the course/velocity setpoints, USV path, translation of the predictions to the occupancy probability grid (estimated future collisions (EFC) and selected future path (SFP)) and Heuristics (51).

In this first stage of development, like other reactive algorithms designed for USVs [14,16,35,36,37,45,47], the RRSOAS was evaluated by means of numerical simulations. The Runge–Kutta numerical integration method of fourth order was used with an integration step $T_{s}=0.01$ s. As USV, the set formed by the following models has been used: vessel (1), actuators (3) and course/velocity controllers (5). In addition, the information of the environment that surrounds the USV was generated by the LIDAR sensor model (7) and translated to an occupancy probability grid by the Bayesian filter (15). In turn, the reactive algorithm ran with a sample time $T_{m}=1$ s. As a visual support, Figure 11 shows an instant during RRSOAS execution. On the other hand, each parameter of the algorithm has been adjusted, taking into account its physical meaning, in an iterative process in scenario 3; see Figure 3. As a result, Table 1 was obtained. Scenario 3 was chosen to tune the RRSOAS due to the work previously done in [31], where several SOA methods were tuned on this scenario; and we used the guidance of the USV (1) based on the same environmental information (8). Thus, the RRSOAS can be compared with other SOA methods [11,30,31,41] under the same conditions.

### 4.1. Performances Analysis

**Figure 12:**
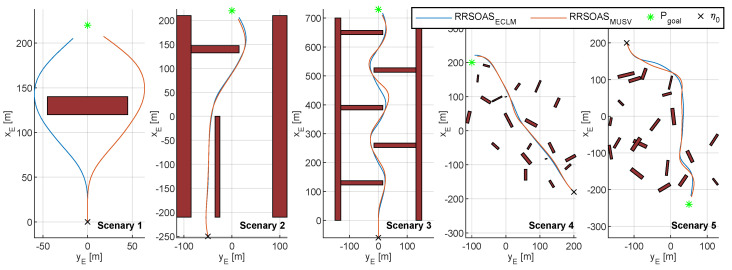
A comparative of the RRSOAS operating with two prediction models: ECLM (28) and USVM—(1), (3), (4) and (5). The adjustment parameters used can be found in Table 1.

First, the performance of the RRSOAS was evaluated when it used the new ECLM (28) as an estimator of future paths. To that end, this performance was compared with the one that would be obtained if, when making the predictions, the USV’s dynamics was fully known. This analysis would not be possible in real sea trials, since all mathematical modeling presents different kinds of uncertainties that deviate it from the real system’s behavior [66]. However, in numerical simulations, it is possible to use the same dynamic model that represents the behavior of the USV as a prediction model. This approach was used in [16,17,34,36,39,40,44,46], where the authors also validated OA systems through numerical simulations and the only uncertainties considered in the prediction model were the ones associated with the effects that external disturbances caused on the vessel. In line with these works, the USV model (Equations (1) and (3)–(5), USVM) was discretized by using the Euler forward method. Thus, the performance of the RRSOAS was compared with two different prediction models: the USVM and the ECLM; in other words, two reactive algorithms were compared: RRSOAS_USVM_ and RRSOAS_ECLM_. Specifically, this comparative was carried out over the five scenarios defined in [31], in which the vehicle started moving at the goal velocity $U_{goal}=u=7$ m/s and the ocean current was set as $V_{c}=1$ kn with direction $\beta_{c}=0$º. As tuning parameters, in both cases the ones listed in Table 1 were used. As a result, Figure 12 shows the paths obtained with the USV’s guidance being carried out by the RRSOAS algorithm with each prediction model. As can be seen, in both cases the algorithm successfully guided the USV to $P_{\mathrm{goal}}$ in the presence of disturbances. Furthermore, with the exception of scenario 1, wherein heuristics (51) generated a different initial decision, the paths followed by the USV did not present significant changes. This is due to the fact that, independently of the fit between the prediction model and the behavior of the USV, RRSOAS takes into account that predictions of future paths will always contain accumulative errors caused by the uncertainty. This feature is modeled through the variable ellipse (43), whose perimeter represents the shape of the vessel; see Figure 9.

In addition to the vessel’s paths, this work quantitatively evaluated the performances in the guidance of the USV. These performances, in line with the approach followed in [31], are defined according to the following indicators: mission duration ($t_{m}$), distance covered during the mission ($d_{m}$) and control effort required (Δc); see Equation (55).

(55) $$\begin{array}{cc} t_{m}\left(k\right) & =kT_{m}\\ d_{m}\left(k\right) & =∥\eta_{l}\left(k\right)-\eta_{l}\left(k-1\right)∥+d_{m}\left(k-1\right),\;\eta_{l}\left(k\right)=\left(x_{E}\left(k\right),\;y_{E}\left(k\right)\right)\\ \Delta c(k) & =\frac{|sp_{\chi }\left(k\right)-sp_{\chi }\left(k-1\right)|}{\pi }+\frac{|sp_{U}\left(k\right)-sp_{U}\left(k-1\right)|}{U_{lim}}+\Delta c\left(k-1\right) \end{array}$$

These indicators have been obtained for the paths shown in Figure 12 and are collected in Table 2. As can be seen, the RRSOAS presents very similar indicators when guiding the USV with both prediction models. Therefore, given the combination of the ECLM (28) with the variable ellipse (43), both novel proposals of this work can be used to carry out the autonomous guidance of vessels, whose mathematical models, and those of their controllers, are unknown. Hence, in all the results shown below, the ECLM was used as a prediction model (RRSOAS = RRSOAS_ECLM_). Moreover, ECLM presents two advantages over the general approach [16,17,34,36,39,40,44,46]. Firstly, it does not depend on previous mathematical modeling of the vessel or its controllers. Secondly, it makes the simulation environment more realistic, since the dynamic model that represents the USV’s behavior is not used as predictive model. Consequently, as would happen on a real USV, the estimation of future paths contemplates prediction errors.

Additionally, here the RRSOAS is compared with two SOA methods. The first method is the LROABRA (local reactive obstacle avoidance based on region analysis) algorithm, which has been specifically designed to provide vessels with the capacity to avoid static obstacles at high navigation velocities [41]. The second SOA method considered is the VFH+, which was proposed in [30] as an improvement of the VFH [51], and later used as an OA system for a USV [11]. Specifically, to carry out the comparison, the results obtained in [31] are used, where both methods were autotuning with a genetic algorithm on scenario 3, and in turn, evaluated over the scenarios shown in Figure 3. These results, which are directly related to the indicators (55), have been included in Table 3 together with the performances obtained by the RRSOAS. Note that, according to the results obtained in [31], the LROABRA algorithm, automatically tuned for scenario 3, does not pass scenario 4. In particular, it can be seen how RRSOAS, with respect to the LROABRA and VFH+ methods, achieves improved mission times and reduced distances covered in most scenarios. As an exception, in scenario 4 the VFH+ method achieves slightly higher performance (it reduces mission time and distance by 1.32% and 3.23%, respectively). Therefore, for the USV (1), using the measurements provided by the LIDAR model (8), the new RRSOAS proposed in this paper offers competitive performance in autonomous guidance with respect to other SOA methods applied to USVs [11,30,41].

**Figure 13:**
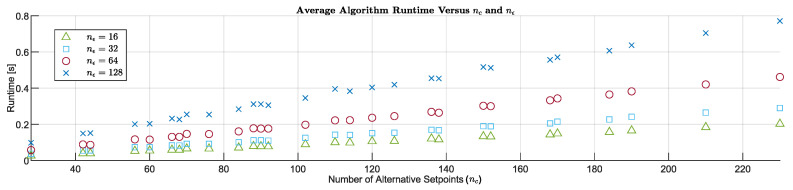
Variation of average RRSOAS runtimes as a function of the number of alternative government setpoints ($n_{c}$) and the number of points that model the shape of the USV ($n_{\epsilon }$).

Continuing with the analysis of the RRSOAS, two new proposals have been presented in this paper with respect to the algorithm’s runtime. The first one is focused on the effective discretization of the decision space (16), by using an exponential resolution (17) in the course setpoints. In addition, the prediction horizon (37) for each possible future path that the USV could follow is variable. Since the final objective of an OA system is its implementation in a real vehicle, it is necessary to ensure that its computation is feasible between periods of execution of the same one. Due to the establishment times associated with the marine surface vehicle [5,57,58,59,60], sample times in reactive algorithms applied to USVs are generally higher than those used for other kinds autonomous vehicles [20,21,61,62]. As an example, the authors in [13,15,17,56] established sample times between 1 and 2.5 seconds for their reactive algorithms, which were evaluated with vessels similar to the USV (1) by means of numerical simulations in [13,15,17,56] and on real vessels in [13,17]. In other works [16,34], where the reactive algorithms were evaluated through numerical simulations, the sample times were 5 seconds (for a 32-meter vessel [34]). With regard to the RRSOAS, Figure 13 shows how the algorithm’s runtime varies (average of 100 iterations) depending on the number of alternative government setpoints ($n_{c}$) and the number of points ($n_{\epsilon }$) used to discretize ellipse (40). These times have been measured from MATLAB’s *Tic-Toc* functions [77], while the algorithm is running in Simulink on a laptop model *msi GT60 2PC Dominator* [78]. As can be seen, for the nominal adjustment shown in Table 1 ($n_{c}=76$ and $n_{\epsilon }=32$), the average runtime is 0.092 s. Moreover, for the limits considered in this work to implement the algorithm ($n_{c}=230$ and $n_{\epsilon }=128$), an average runtime lower than the sample ($T_{m}=1$ s) time is obtained (0.771 s). Therefore, if a hardware platform with similar computing power is used, it would be feasible to implement the new RRSOAS in a real vessel. In addition, it should be noted that these times could be improved if the algorithm ran on a hardware platform completely dedicated to it.

### 4.2. Robustness Analysis

Obstacle avoidance methods are adjusted on specific scenarios [12], which does not ensure that the OA system can overcome all situations [31]. This is due to the infinite geometric and environmental combinations that define the possible scenarios in which a USV could navigate. In this sense, as a novel contribution of this work, we propose to evaluate the robustness of the reactive algorithm on a sample of random scenarios. To this end, a sample of one-hundred scenarios has been generated following the procedure described in Section 2.3. $\Theta_{E}=\left(100\;20\;300\;60\;20\right)$ has been set as the characteristic vector of the sample. In addition, this sample is parameterized in terms of the goal velocity ($U_{goal}$) and the current velocity ($V_{c}$). Thus, it is possible to study the reactive algorithm in several USV’s operation points, under the effect of different levels of environmental disturbances and the presence of random distributions of obstacles. Specifically, the sample of random scenarios is parameterized for nine different situations, which are obtained from the combination of $U_{goal}=\left\{5\;7\;9\right\}$ m/s and $V_{c}=\left\{0.5\;1\;2\right\}$ kn. Note that, by combining the above sets with the sample of one-hundred scenarios, the RRSOAS is evaluated over nine-hundred different scenarios. This study includes the following performance indicators: success rate, stop rate, collision rate, average mission time $\left(\bar{t_{m}}\right)$, average distance traveled $\left(\bar{d_{m}}\right)$ and average control effort $\left(\bar{\Delta c}\right)$. In the success rate, the guidance is correct if the vehicle reaches $P_{\mathrm{goal}}$ without suffering any collision. During the simulations, the collision situation will occur if the distance between the USV and an obstacle is less than $L_{USV}/2$. On the other hand, the stop condition will occur if the reactive algorithm holds a zero velocity setpoint for a time $t_{stop}=10$ s, whenever a collision situation has not previously occurred. This time has been set based on the braking response of the USV (1) in closed-loop (5). Finally, from the arithmetic mean of the indicators (55), the values $\left(\bar{t_{m}},\;\bar{d_{m}},\;\bar{\Delta c}\right)$ are calculated for those scenarios where the USV is successfully guided to $P_{\mathrm{goal}}$. In addition, as a complementary result of this work, a more conservative tuning of the RRSOAS has been done in order to show its capacity to increase the USV’s safety. Therefore, the RRSOAS was studied with the performance tuning of Table 1; RRSOAS^PT^, and with the conservative tuning; RRSOAS^CT^. In this conservative tuning, with respect to the parameters listed in Table 1, the safety factors that oversize the vessel have been increased ($\gamma_{L}=1.5$, $\gamma_{M}=3$, $\gamma_{v}=4.5$), the algorithm’s behavior has been modified to avoid leading the USV towards occupied zones ($T_{mac}=30$, $\alpha_{1}=0.4$, $\alpha_{3}=1$, $\alpha_{4}=0.2$) and the number of alternative velocity setpoints has been increased ($n_{u}=2$). Note that, in both adjustments, the RRSOAS uses the ECLM (28) as the prediction model. Finally, in order to compare the robustness of RRSOAS with another reactive method, VFH+ is also evaluated in the same random scenarios. In particular, the VFH+ (version available in Simulink [76]) is chosen because it is the most robust of the SOA methods [30,35,41,42] studied in [31].

As results, Table 4 presents the indicators obtained by the reactive algorithms for each sample of scenarios parameterized in terms of $U_{goal}$ and $V_{c}$. First, a comparative between VFH+ and RRSOAS^PT^ is made, taking into account that both have been adjusted on the same scenario. As can be seen, the two algorithms present similar success rates for $U_{goal}=5$ m/s. However, as an advantage of the RRSOAS^PT^, its collision rate is much lower (0.67% versus 12%). On the other hand, for $U_{goal}=7$ m/s and $U_{goal}=9$ m/s, the RRSOAS^PT^ success rate is much higher than VFH+ (92.83% versus 83.17%), as is its capacity to avoid collisions (99% versus 91%). Moreover, in terms of average performance indicators, RRSOAS^PT^ outperforms VFH+ in most scenarios. As an exception, in scenarios where $U_{goal}=9$ m/s, the distance traveled by the USV is less under the government of the VFH+. With regard to the RRSOAS^PT^ and RRSOAS^CT^ adjustments, a more conservative tuning increases the robustness of the algorithm and USV’s safety, but causes a loss of performance in most cases.

As a summary, Table 5 shows the average of all the indicators listed in Table 4. As can be seen, the RRSOAS outperforms all the average performances obtained by the VFH+. Furthermore, with a more conservative tuning, the success rate of the RRSOAS has been significantly increased (94.33% versus 86.78%), and over the nine-hundred scenarios analyzed, the vessel did not suffer any collisions. It should be noted that these results do not guarantee that the RRSOAS can overcome any static obstacle scenario. Although, these results demonstrate the viability of implementing and evaluating the new RRSOAS on a real USV.

**Figure 14:**
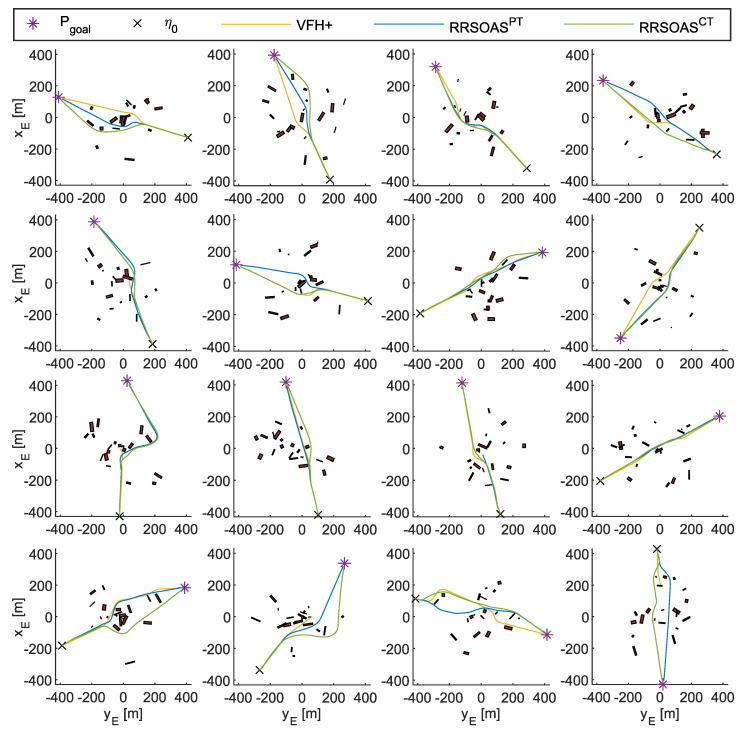
Paths of the vessel (1), in sixteen of the nine-hundred scenarios studied in this work, when it is guided autonomously by the algorithms RRSOAS and VFH+ [30,76].

Finally, as a visual report, Figure 14 shows the paths followed by the USV in sixteen of the nine-hundred scenarios analyzed in this paper. In these scenarios the USV was guided by the VFH+ and RRSOAS algorithms, where the RRSOAS was tuned in two different ways: conservative tuning and performance tuning.

## 5. General Discussion

Due to the current interest in the field of USVs, this paper proposes the new Robust Reactive Static Obstacle Avoidance System (RRSOAS) and used computing simulations to test our proposal. In terms of performance, a comparison of the new RRSOAS with other SOA methods applied to the USV has been carried out (VFH+ [11,30] and LROABRA [41]). The obtained results show how, in most cases, the RRSOAS outperforms these reactive algorithms. Furthermore, for several points of operation of the USV, a robustness study of the RRSOAS has been carried out on nine-hundred random scenarios in the presence of different current levels. In this aspect, the RRSOAS is significantly more robust than the VFH+, and with a conservative tuning, it guarantees the safety of the USV. In addition, the algorithm’s runtime is considerably less than its sample time. Therefore, its implementation and evaluation in a real USV are feasible and proposed as future work. Moreover, taking into account these results, several of RRSOAS’s contributions could be integrated into other reactive algorithms applied to USVs. Firstly, RRSOAS does not depend on a priori knowledge of a mathematical model of the vessel and its controllers for velocity and course. Instead, we propose to use an estimated closed-loop model (ECLM) of the USV, which is identified from time series in which an experienced pilot carries out obstacle avoidance maneuvers. This ECLM could also be used by other reactive methods that make predictions of possible future paths of the vehicle [16,17,20,34,36,39,40,44,46], thus eliminating the dependence on USV’s mathematical modeling that the use of such methods implies. In addition, with the aim of considering the prediction errors due to uncertainty, these works could integrate the variable ellipse with the prediction step proposed in this paper to model the USV’s shape. On the other hand, an occupancy probability grid is used by RRSOAS. In this way, the reactive algorithm contemplates the errors and measurement uncertainty of the sensors. Another novel contribution has been made in this aspect; the repulsive forces proposed in [29] have been adapted in order to consider the USV’s dynamics and the probability of occupation. These repulsive forces can also be applied to other OA systems for USVs based on potential fields [32,35,38,42,45]. Furthermore, in order to limit the algorithm’s runtime, a variable horizon is proposed for the future path predictions, along with an exponential discretization to generate the alternative course setpoints. This last contribution of our method, given the successful results obtained in the numerical simulations, could be applied to other reactive algorithms which require discrete direction commands [13,14,16,17,26,27,30,41]. Finally, in order to take into account dynamic obstacles, the RRSOAS could easily be combined (since it does not need to know the model of the vessel or its controllers) with reactive algorithms designed to avoid dynamic obstacles according to the COLREGS regulation [13,14,16,36,39,40,46].

## 6. Conclusions

According to the results obtained in the numerical simulations carried out in this work, it is possible to conclude that the new RRSOAS is robust to unknown and congested scenarios in the presence of disturbances, while offering very competitive performance with respect to other reactive algorithms previously applied to USVs. In addition, based on the RRSOAS’s runtimes, it could be implemented and evaluated in a real USV as a static obstacle avoidance system. Moreover, for its operation, this reactive algorithm only needs as inputs: the state vector of the USV, an occupancy probability grid and goal setpoints. In particular, the RRSOAS is the result of the integration of several novel contributions with regard to the current state-of-the-art of USVs that we proposed in this work. Firstly, the algorithm generates a new effective discrete decision space from an exponential resolution applied to the course setpoints; this resolution is proposed in our work. Secondly, a new estimated closed-loop model is proposed in this paper, which is used to make predictions of the possible future paths that the USV could follow if a collision risk were to arise. Thirdly, these paths are translated into an occupancy probability grid, where each path is characterized by taking into account the USV’s dynamics, the uncertainty present in the prediction model and the occupancy probability. Finally, a heuristic system designed specifically for USVs is used to calculate the course and velociy setpoints. These setpoints are the RRSOAS’s outputs, which are demanded by the USV’s controllers.

## Figures and Tables

**Table 1:** RRSOAS parameters in international units, which have been tuned in scenario 3 of Figure 3.

Navigation Behaviour		Safety and Sizing		Path Predictor	Discrete Decision Space	
$\alpha_{1}=0.5$ $\alpha_{3}=0.7$	$\alpha_{2}=0.3$ $\alpha_{4}=0.25$	$T_{mac}=20$	$\gamma_{L}=1.25$	$D_{p}=200$	$n_{U}=1$	$U_{lim}=10$
		$\gamma_{v}=3.4$	$\gamma_{M}=2.45$	$T_{p}=0.1$	$n_{\chi }=9$	$\Delta \chi_{max}=\pi /2$
		$n_{t}=0.75$	$n_{\epsilon }=32$	$M_{max}=1100$	$\tau_{\chi }=2.2$	$\kappa_{\chi }=0.1$

**Table 2:** Indicators (55) achieved by RRSOAS algorithm with each prediction model in the scenarios proposed in [31]. These indicators characterize the paths shown in Figure 12.

Methods	Scenario 1			Scenario 2			Scenario 3			Scenario 4			Scenario 5		
	$t_{m}$	$d_{m}$	Δc	$t_{m}$	$d_{m}$	Δc	$t_{m}$	$d_{m}$	Δc	$t_{m}$	$d_{m}$	Δc	$t_{m}$	$d_{m}$	Δc
RRSOAS_ECLM_	38	239	0.9	73	733	1.1	123	890	1.8	76	776	1.0	79	746	2.3
RRSOAS_MUSV_	39	246	0.9	72	729	0.6	125	904	2.0	76	770	1.2	79	753	1.7

**Table 3:** Indicators (55) achieved by each obstacle avoidance method in the scenarios proposed in [31]. The results of the VHF+ [30,76] and LROABRA [41] methods have been obtained in [31].

Methods	Scenario 1			Scenario 2			Scenario 3			Scenario 4			Scenario 5		
	$t_{m}$	$d_{m}$	Δc	$t_{m}$	$d_{m}$	Δc	$t_{m}$	$d_{m}$	Δc	$t_{m}$	$d_{m}$	Δc	$t_{m}$	$d_{m}$	Δc
RRSOAS_ECLM_	38	239	0.9	73	733	1.1	123	890	1.8	76	776	1.0	79	746	2.3
LROABRA [41]	69	346	2.4	82	752	0.9	145	929	2.2	—	—	—	88	758	1.5
VFH+ [30,76]	47	268	1.4	78	744	0.8	148	950	2.6	75	751	1.0	92	794	1.8

**Table 4:** Indicators obtained by the reactive algorithms for each sample of one-hundred random scenarios parameterized in terms of $U_{goal}$ and $V_{c}$.

Characterization of Random Scenarios	Algorithm	*Success* [%]	*Stop* [%]	*Collision* [%]	$\bar{t_{m}}$ [s]	$\bar{d_{m}}$ [m]	$\bar{\Delta c}$
$U_{goal}=5$ m/s $V_{c}=0.5$ kn	VHF+ $RRSOAS^{PT}$ $RRSOAS^{CT}$	79 79 95	11 20 05	10 01 00	181.3 174.3 178.6	1247 1234 1252	2.36 1.93 1.97
$U_{goal}=5$ m/s $V_{c}=1$ kn	VHF+ $RRSOAS^{PT}$ $RRSOAS^{CT}$	72 73 92	13 26 08	15 01 00	181.2 173.6 177.6	1247 1231 1248	2.24 1.90 1.92
$U_{goal}=5$ m/s $V_{c}=2$ kn	VHF+ $RRSOAS^{PT}$ $RRSOAS^{CT}$	76 72 88	13 28 22	11 00 00	182.1 173.7 178.1	1249 1231 1251	2.30 1.90 1.94
$U_{goal}=7$ m/s $V_{c}=0.5$ kn	VHF+ $RRSOAS^{PT}$ $RRSOAS^{CT}$	85 91 95	08 18 05	07 01 00	131.7 128.1 130.0	1232 1231 1235	2.31 1.94 1.84
$U_{goal}=7$ m/s $V_{c}=1$ kn	VHF+ $RRSOAS^{PT}$ $RRSOAS^{CT}$	82 90 97	11 09 03	07 01 00	131.4 128.1 131.1	1231 1231 1241	2.30 1.96 1.88
$U_{goal}=7$ m/s $V_{c}=2$ kn	VHF+ $RRSOAS^{PT}$ $RRSOAS^{CT}$	86 90 94	10 09 06	04 01 00	132.1 127.5 130.5	1233 1229 1241	2.39 1.93 1.88
$U_{goal}=9$ m/s $V_{c}=0.5$ kn	VHF+ $RRSOAS^{PT}$ $RRSOAS^{CT}$	81 96 96	07 03 04	12 01 00	103.0 101.3 103.8	1209 1221 1234	2.29 1.82 1.92
$U_{goal}=9$ m/s $V_{c}=1$ kn	VHF+ $RRSOAS^{PT}$ $RRSOAS^{CT}$	82 95 97	06 04 03	12 01 00	103.4 101.5 104.6	1212 1220 1239	2.27 1.84 1.96
$U_{goal}=9$ m/s $V_{c}=2$ kn	VHF+ $RRSOAS^{PT}$ $RRSOAS^{CT}$	83 95 95	05 04 05	12 01 00	103.5 101.0 105.1	1211 1221 1242	2.32 1.82 2.00

**Table 5:** Average indicators for the nine-hundred scenarios shown in Table 4.

Algorithm	*Success* [%]	*Stop* [%]	*Collision* [%]	$\bar{t_{m}}$ [s]	$\bar{d_{m}}$ [m]	$\bar{\Delta c}$
VHF+ $RRSOAS^{PT}$ $RRSOAS^{CT}$	80.67 86.78 94.33	09.33 12.33 05.67	10.00 00.89 00.00	138.9 134.4 137.7	1230 1228 1243	2.31 1.89 1.92
